# A Longitudinal Study on the Dynamics of Salmonella enterica Prevalence and Serovar Composition in Beef Cattle Feces and Lymph Nodes and Potential Contributing Sources from the Feedlot Environment

**DOI:** 10.1128/aem.00033-23

**Published:** 2023-04-06

**Authors:** Colette Nickodem, Ashley N. Arnold, Kerri B. Gehring, Jason J. Gill, John T. Richeson, Kendall L. Samuelson, H. Morgan Scott, Jason K. Smith, T. Matthew Taylor, Javier Vinasco, Keri N. Norman

**Affiliations:** a Department of Veterinary Integrative Biosciences, Texas A&M University, College Station, Texas, USA; b Texas A&M Veterinary Medical Diagnostic Laboratory, Texas A&M University, College Station, Texas, USA; c Department of Animal Science, Texas A&M University, College Station, Texas, USA; d Center for Phage Technology, Texas A&M University, College Station, Texas, USA; e Department of Agricultural Sciences, West Texas A&M University, Canyon, Texas, USA; f Department of Veterinary Pathobiology, Texas A&M University, College Station, Texas, USA; g Texas A&M AgriLife Extension, Department of Animal Science, Texas A&M University, Amarillo, Texas, USA; INRS Armand-Frappier Sante Biotechnologie Research Centre

**Keywords:** *Salmonella enterica*, feedlot cattle, feedlot pen environment, subiliac lymph nodes

## Abstract

Salmonella can persist in the feedlot pen environment, acting as a source of transmission among beef cattle. Concurrently, cattle that are colonized with Salmonella can perpetuate contamination of the pen environment through fecal shedding. To study these cyclical dynamics, pen environment and bovine samples were collected for a 7-month longitudinal comparison of Salmonella prevalence, serovar, and antimicrobial resistance profiles. These samples included composite environment, water, and feed from the feedlot pens (*n* = 30) and cattle (*n* = 282) feces and subiliac lymph nodes. Salmonella prevalence across all sample types was 57.7%, with the highest prevalence in the pen environment (76.0%) and feces (70.9%). Salmonella was identified in 42.3% of the subiliac lymph nodes. Based on a multilevel mixed-effects logistic regression model, Salmonella prevalence varied significantly (*P* < 0.05) by collection month for most sample types. Eight Salmonella serovars were identified, and most isolates were pansusceptible, except for a point mutation in the *parC* gene, associated with fluoroquinolone resistance. There was a proportional difference in serovars Montevideo, Anatum, and Lubbock comparing the environment (37.2, 15.9, and 11.0%, respectively), fecal (27.5, 22.2, and 14.6%, respectively), and lymph node (15.6, 30.2, and 17.7%, respectively) samples. This suggests that the ability of Salmonella to migrate from the pen environment to the cattle host—or vice versa—is serovar specific. The presence of certain serovars also varied by season. Our results provide evidence that Salmonella serovar dynamics differ when comparing environment and host; therefore, developing serovar-specific preharvest environmental Salmonella mitigation strategies should be considered.

**IMPORTANCE** Salmonella contamination of beef products, specifically from the incorporation of bovine lymph nodes into ground beef, remains a food safety concern. Current postharvest Salmonella mitigation techniques do not address Salmonella bacteria that are harbored in the lymph nodes, nor is it well understood how Salmonella invades the lymph nodes. Alternatively, preharvest mitigation techniques that can be applied to the feedlot environment, such as moisture applications, probiotics, or bacteriophage, may reduce Salmonella before dissemination into cattle lymph nodes. However, previous research conducted in cattle feedlots includes study designs that are cross-sectional, are limited to point-in-time sampling, or are limited to sampling of the cattle host, making it difficult to assess the Salmonella interactions between environment and hosts. This longitudinal analysis of the cattle feedlot explores the Salmonella dynamics between the feedlot environment and beef cattle over time to determine the applicability of preharvest environmental treatments.

## INTRODUCTION

Salmonella infects 1.35 million people in the United States each year, with an estimated 1 million cases attributed to contaminated food products (1). Nontyphoidal salmonellosis outbreaks in the United States can result from the consumption of contaminated meat, such as beef, pork, and poultry products, leafy greens, nut spreads, and other processed foods. The Centers for Disease Control and Prevention (CDC) FoodNet FAST population survey estimates that 82.2% of Americans consume beef products on a weekly basis, and more specifically, 67% consume ground beef (2). Considering the high rate of beef consumption, an estimated 10% of salmonellosis cases can be attributed to beef products in the United States, although this is lower than estimates for pork or poultry products (3).

There are over 2,500 serovars of Salmonella, which are distinguished by surface antigens (4, 5). Different Salmonella serovars can have a range of characteristics that contribute to host specificity, pathogenicity, and antimicrobial resistance profiles (6). The CDC reports that the four most commonly identified Salmonella serovars related to human infection are Enteritidis, Typhimurium, Newport, and Javiana (7). The most prevalent serovars of Salmonella identified in cattle around the world are Montevideo, Typhimurium, Anatum, Kentucky, Newport, Cerro, Mbandaka, and Muenster (8). Serotyping assists with tracking Salmonella foodborne outbreaks from infected humans to the source. Importantly, a recent outbreak in 2018 to 2019 reported 403 infections and 117 hospitalizations across 30 states caused by antibiotic-resistant Salmonella Newport; further, outbreak investigations continue to implicate ground beef products (9). Salmonella is considered a serious threat for the continued emergence of antimicrobial resistance (AMR), with an estimated 40% increase of resistance in Salmonella from human infections during 2015 to 2016 compared to 2004 to 2008 (10). Tracking Salmonella serovars of public health importance, or with clinically relevant AMR profiles, will help to identify harmful sources in different food and animal production systems.

Despite standard postharvest interventions during beef processing, such as evisceration and organic acid applications, there has been little reduction in Salmonella contamination of ground beef products in recent years (11, 12). For example, in 2014 the Food Safety and Inspection Service (FSIS) estimated Salmonella prevalence in ground beef to be 1.6%; however, in 2021, FSIS identified Salmonella in 2.25 to 3.77% of raw ground beef (12, 13). Current practices focus on but are not limited to organic acid antimicrobials and thermal (i.e., hot water washes or steam pasteurization) carcass applications to reduce Salmonella contamination after hide removal (14–16). Different application techniques have been tested to determine the most effective strategies to mitigate Salmonella in beef processing plants (17–19). However, there is a lack of postharvest interventions addressing lymph nodes harboring Salmonella. Salmonella can reside in cattle lymph nodes where it is impervious to antimicrobials applied to the carcass surface. Many lymph nodes in beef cattle, notably the subiliac and superficial cervical nodes, are surrounded by adipose tissue located adjacent to meat cuts or trim used for human consumption. These lymph nodes are difficult to separate from the fatty tissues, which are commonly incorporated into ground beef products. Research originally identified Salmonella in cattle mesenteric lymph nodes (20), which have been reported to have a higher prevalence (91.2%) than other lymph node locations (7.4 to 76.5%). However, mesenteric lymph nodes are removed during evisceration, unlike the subiliac and superficial cervical lymph nodes. Recent studies have focused on the peripheral lymph nodes (21–25), but the transmission route and mechanism Salmonella uses to invade the lymph nodes are not well understood, making the development of intervention strategies difficult. Understanding the Salmonella dynamics at cattle feedlots may provide further evidence linking Salmonella in the feedlots to cattle and their lymph nodes. While it is not feasible to address all sources of Salmonella at the feedlot that may contribute to Salmonella in cattle and their lymph nodes, it may be possible to address specific serovars of concern within the pen environment.

Beef cattle originating from different source locations are known to have distinct Salmonella profiles on arrival at the feedlot (26). Asymptomatic Salmonella colonization is common in the intestinal tract of ruminants and contributes to fecal shedding, contaminating the pen environment and ultimately other cattle within the same and adjacent pens (27–29). In addition to the hind gut, Salmonella has been identified in other locations of beef cattle, including multiple hide locations, the oral cavity, and lymph nodes (30). Salmonella has been detected in the pen soil, water troughs, and feed bunks, where it can remain for extended periods of time and be transmitted to other cattle (31–34). For example, Dargatz et al. collected monthly samplings of feed ingredients from December 2001 to November 2002 and identified Salmonella in 5.3% (57/1,070) of feed samples (35). Previous research investigating Salmonella in the feedlot environment, specifically pen soil, is limited. For example, a study conducted by Xie et al. evaluated triplicate single-time-point samples from 3 pens at 3 feedlots (*n* = 27) in Texas, finding that soil and water had the highest prevalence and feces had the lowest prevalence of Salmonella (36). Rodriguez et al. analyzed soil, bedding, feed, and feces samples every 3rd month across 18 months from beef, dairy, poultry, and swine farms from 8 states, but only 8.3% (4/48) of samples collected from beef farm soil were positive for Salmonella (37). Neither study specifically investigated Salmonella in the beef cattle feedlot environment. Comparatively speaking, there are many studies with extended sampling periods of cattle hides and feces (27, 29, 30).

Monitoring longitudinal Salmonella prevalence, serovar composition, and AMR profiles from the feedlot pen and beef cattle may provide better insights into the Salmonella dynamics between environment and host. This research will also determine the relative utility of exploring Salmonella mitigation options within the feedlot environment, such as moisture applications, probiotics, or bacteriophage.

## RESULTS

### Quantification of samples.

A total of 1,654 samples were collected during the study period, comprised of 752 freshly voided feces, 192 pen environment-manure pack, 188 pen environment-dry, 146 pen trough water, 149 feed, and 227 pooled cattle subiliac lymph node samples (see Table S1 in the supplemental material). One feed sample was missed during the October collection from pen 49. Lymph nodes were not collected from the first set of cattle (*n* = 40; pens 57 to 60) that went to slaughter, and there were an additional 15 unobtained lymph node samples that either were lost during collection or the cattle died prior to slaughter.

### Salmonella prevalence by sample type.

**FIG 1:**
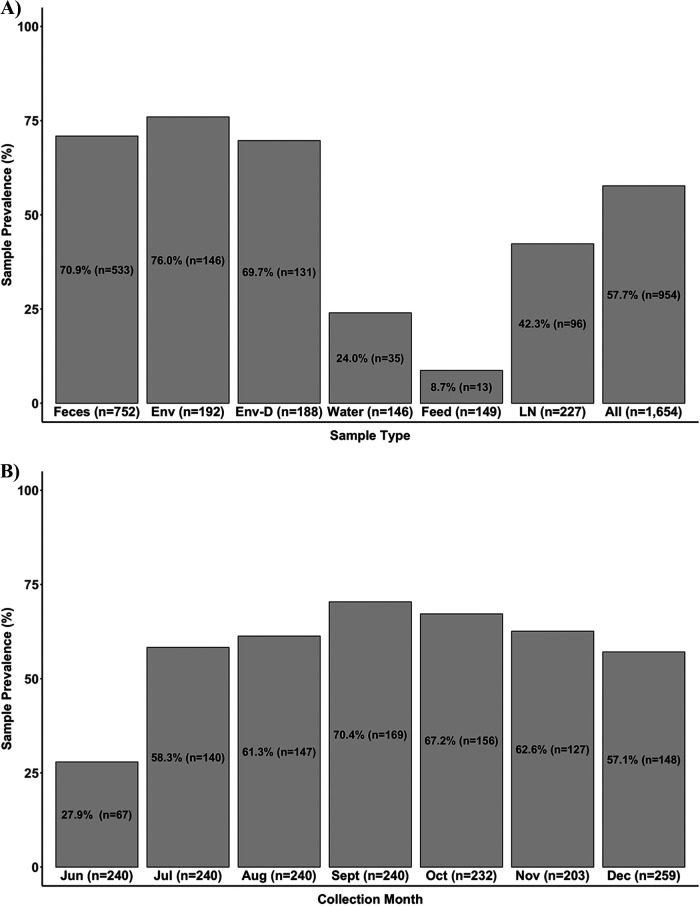
(A) Salmonella prevalence (percentage, number of positive samples) by sample type (Env, pen environment-manure pack; Env-D, pen environment-dry; LN, lymph node; All, all sample types) with the total number of each sample type provided in the parentheses below each bar. The bar farthest to the right provides the overall Salmonella prevalence for all samples. (B) Salmonella prevalence by collection month with the number of total samples per month provided in parentheses below each bar.

Salmonella prevalence was 57.7% (954/1,654) among all sample types collected (Fig. 1A). The highest prevalence was found in pen environment-manure pack (76.0%, 146/192), closely followed by freshly voided feces (70.9%, 533/752) and pen environment-dry (69.7%, 131/188). Pen trough water (24.0%, 35/146) and feed (8.7%, 13/149) prevalences were substantially lower. Importantly, the overall Salmonella prevalence for bovine subiliac lymph nodes was 42.3% (96/227). The first lymph node harvest in October had 100% (17/17) prevalence, which decreased at the second harvest in November (37.3%, 22/59) and remained similar for the third harvest in December (37.8%, 57/151).

### Salmonella prevalence by collection month.

The lowest monthly prevalence of Salmonella from all sample types across the study was during the first sample collection in June (27.9%, 67/240) (Fig. 1B). The highest prevalence was during the early fall: September (70.4%, 169/240), October (67.2%, 156/232), and November (62.6%, 127/203). This was followed closely by the summer months: August (61.3%, 147/240) and July (58.3%, 140/240). Salmonella prevalence remained high during December (57.1%, 148/259). Specifically, for pen environment-manure pack samples, the highest prevalence was observed from September (96.7%, 29/30) through December (94.4%, 17/18). Prevalence was lower for pen environment-dry samples from September (70.0%, 21/30) through December (77.8%, 14/18), with the highest prevalence in October (88.5%, 23/26).

### Salmonella prevalence multilevel mixed-effects logistic regression model.

A multilevel mixed-effects logistic regression model was used to assess Salmonella prevalence by individual sample type (Table S3). For fecal samples, Salmonella prevalence varied significantly for all collection months (*P* < 0.001) compared to the initial collection month. For the forced dietary treatment interaction term, cattle receiving wet distillers’ grains with solubles (WDGS) had 2.12 (*P* = 0.026)-fold-greater odds of Salmonella presence in their fecal samples than did the control group. There was no significant difference in the odds of Salmonella detection (odds ratio [OR] = 1.09, *P* = 0.801) for cattle fed Sweet Bran (SB) compared to the control group.

**FIG 2:**
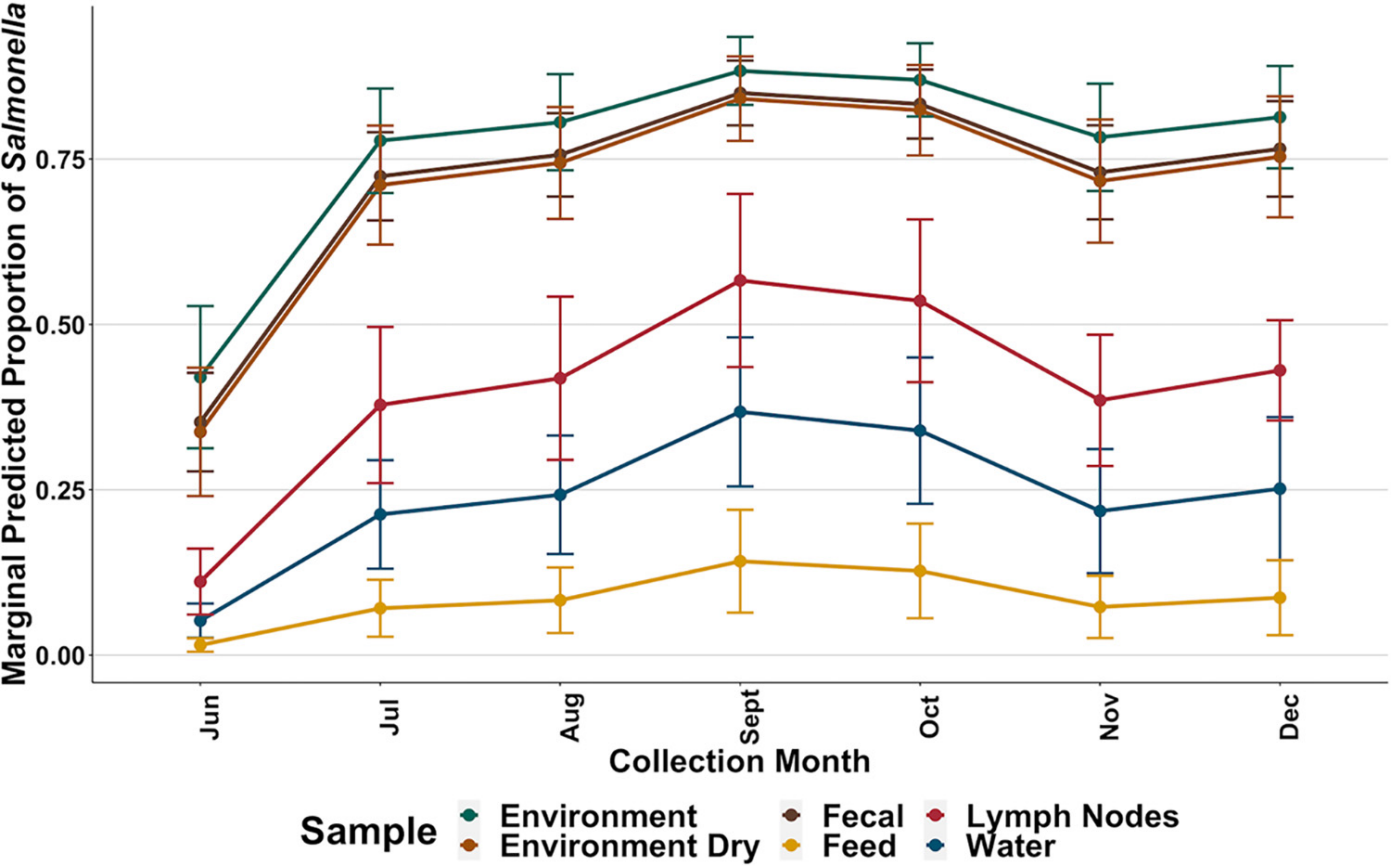
Predictive margin plot from a multilevel mixed-effects logistic regression model of Salmonella prevalence by collection month and sample type, indicated by color as shown in the figure legend.

Within pen environment-manure pack samples, Salmonella prevalence varied significantly (*P* < 0.05) from September through December compared to the initial collection month of June. Interestingly, in pens that cattle were fed SB, the odds of Salmonella detection were reduced (OR = 0.26, *P* = 0.020) for pen environment-manure pack samples, and there was no significant difference in odds (OR = 0.88, *P* = 0.829) for pens in which cattle were fed WDGS compared to the control group. However, this was not observed in the pen environment-dry samples (SB, OR = 0.711, *P* = 0.560; WDGS, OR = 1.41, *P* = 0.570). The analytical model was increasingly unstable for all other sample types due to decreasing sample size; however, predictive margins were compiled, and trends can be observed for all sample types across collection months in Fig. 2.

### Sequencing quality.

There was a total of 524 Salmonella isolates sequenced for this study; of these, 459 arose from the longitudinal analysis, with an additional 30 representing the paired sample data and another 35 included for the phylogenetic analyses. Overall average coverage depth was 38×. The average genome size was 4,809,950 bp, average GC content was 52%, and average number of contigs per assembly was 123.

### Quantification of Salmonella serovars.

Of the 459 isolates that were sequenced as part of the longitudinal study, there were 8 different Salmonella serovars identified. Montevideo (28.5%, *n* = 131), Kentucky (28.3%, *n* = 130), Anatum (20.3%, *n* = 93), Lubbock (12.6%, *n* = 58), and Cerro (8.9%, *n* = 41) were the most frequent. Salmonella serovars; Virginia (0.8%, *n* = 4), Derby (0.2%, *n* = 1), and Senftenberg (0.2%, *n* = 1) were less common. All serovars represented a single sequence type (ST) as follows: Montevideo, ST 138; Kentucky, ST 198; Anatum, ST 64; Lubbock, ST 413; Cerro, ST 367; Virginia, ST 83; Derby, ST 40; and Senftenberg, ST 14.

### Salmonella serovars by sample type.

**FIG 3:**
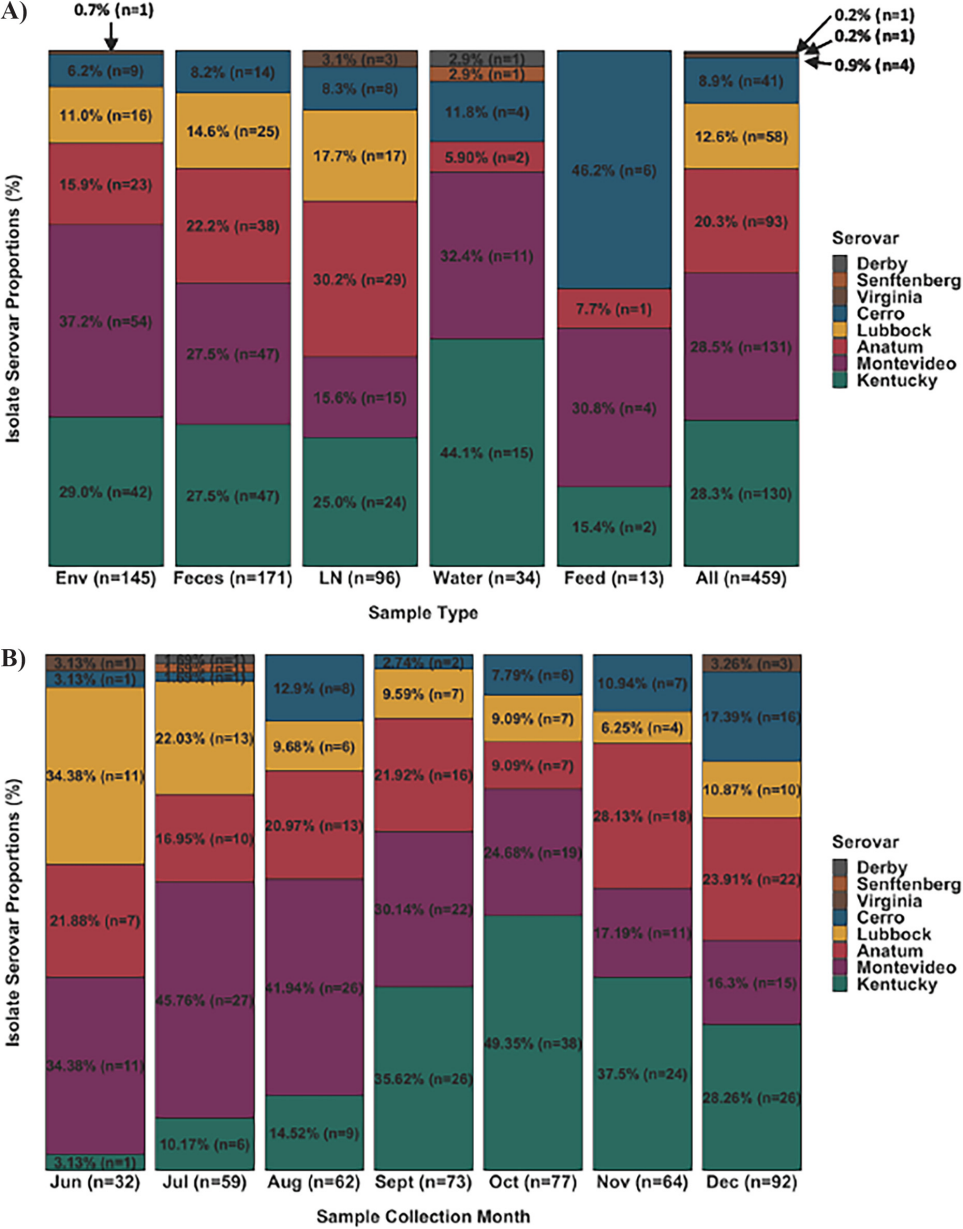
(A) Salmonella serovar composition (percentage, number of isolates) by sample type (Env, environment-manure pack; LN, lymph nodes; All, all sample types) with the furthest bar on the right representing the overall serovar proportions across all sample types and collection months. Serovar is represented by color as shown in the legend. (B) Salmonella serovar composition by collection month.

Proportions of Salmonella serovars varied between environment and host samples (Fig. 3A). Salmonella serovar composition by sample type showed a larger proportion of Montevideo isolates arising from pen environment-manure pack (37.2%, 54/145) than from cattle feces (27.5%, 47/171) and lymph nodes (15.6%, 15/96). There was a smaller proportion of both Lubbock and Anatum isolates arising from pen environment-manure pack (11.0%, 16/145; 15.9%, 23/145, respectively) than from cattle feces (14.6%, 25/171; 22.2%, 38/171, respectively) and lymph nodes (17.7%, 17/96; 30.2%, 29/96, respectively). All other Salmonella serovars did not show a recognizable difference in proportion when comparing environment and host samples; that said, Kentucky exhibited a consistently high prevalence in all three sample types. Isolates from water samples were primarily serovars Kentucky (44.1%, 15/34) and Montevideo (32.4%, 11/34). The majority of feed isolates were of serovars Cerro (46.2%, 6/13) and Montevideo (30.8%, 4/13).

### Salmonella serovars by collection month.

As shown in Fig. 3B, Montevideo and Lubbock were the two serovars most frequently identified in June (34.4%, 11/32 each). Montevideo was the most common serovar in July (45.8%, 27/59) and August (41.9%, 26/62). Salmonella serovar Kentucky was the most frequently observed serovar in the fall months: September (35.6%, 26/73), October (49.4%, 38/77), November (37.5%, 24/64), and December (28.3%, 26/92). Salmonella serovar Anatum contributed to approximately 20% of isolates in all months except for October (9.1%, 7/77). Cerro was not commonly identified compared to other serovars; however, prevalence increased throughout the fall: September (2.7%, 2/73), October (7.8%, 6/77), November (10.9%, 7/64), and December (17.4%, 16/92).

### Salmonella serovar multinomial logistic regression model.

A multinomial logistic regression model was used to assess sample type and serovar associations with serovar Montevideo as the base outcome (Table S4). The subiliac lymph nodes had higher odds of harboring Salmonella serovar Lubbock (OR = 5.06, *P* = 0.029) and Anatum (OR = 2.01, *P* = 0.180) than the base serovar; however, the latter was not significant. In comparison to the base sample type (feces), there were significantly lower odds of Salmonella serovar Anatum recovery from pen environment-manure pack (OR = 0.48, *P* = 0.032) or water samples (OR = 0.19, *P* = 0.05), suggesting Montevideo would be more prevalent in these feedlot pen sample types. Water (OR = 4.88, *P* = 0.05) and feed (OR = 12.69, *P* = 0.005) isolates had significantly higher odds and lymph node (OR = 0.26, *P* = 0.05) isolates had significantly lower odds of being contaminated with Salmonella serovar Cerro than the base sample type. The multinomial nature of the model did not allow for predictive margins; instead, multiple correspondence analysis plots were conducted for visualization.

**FIG 4:**
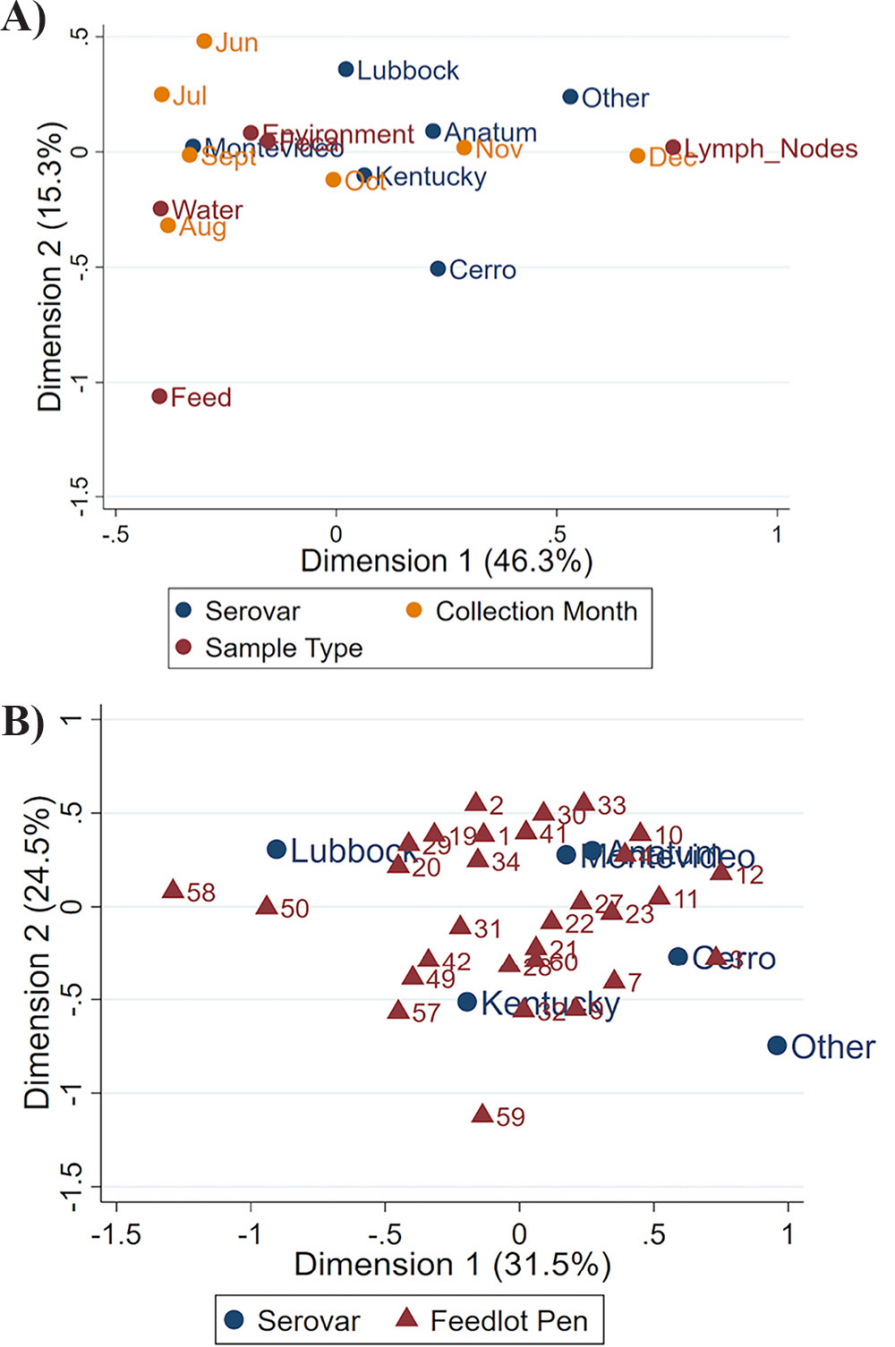
(A) Multiple correspondence analysis (MCA) plot depicting the relationships of serovar and both sample type and collection month; these can be interpreted based on distances and angles from the plot origin. (B) Correspondence analysis plot investigating pen clustering relationships between Salmonella serovars and feedlot pen number.

For multiple correspondence analysis, Burt’s matrix approach was used with principal normalization. Inertia was 61.6% for serovar, sample type, and collection month (Fig. 4A). To determine clustering of serovars by feedlot pen, a multiple correspondence analysis plot was created with an inertia of 56.0% (Fig. 4B).

### Paired samples.

**FIG 5:**
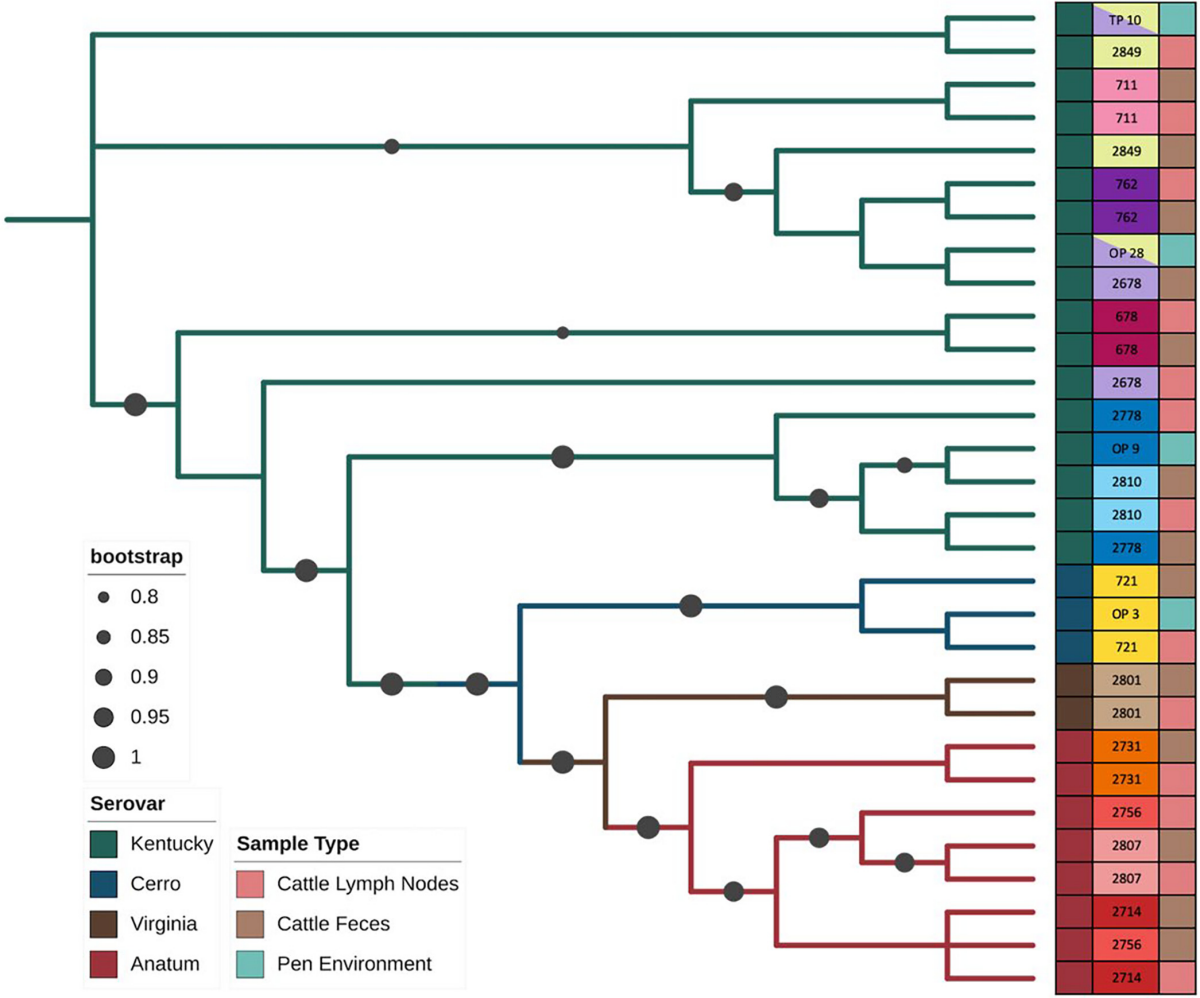
Phylogenetic tree of Salmonella isolates from lymph nodes and terminal fecal samples paired by cattle identifier. If available, pen environment-manure pack isolates (OP, original pen; TP, terminal pen) were matched to the isolates from cattle samples, signified by corresponding colors in the middle column. There could be more than one animal associated with a pen sample, as seen in TP 10 and OP 28, which had two cattle (2678 and 2849) associated with them. The bootstrap values are limited to values 0.8 to 1.0.

There were 49 sequenced pairs of lymph node and fecal isolates from the same animals, and in 38 of those pairs the fecal samples were collected in the terminal month along with the lymph node (Fig. S1). Overall, 28.6% (14/49) of the total paired samples shared the same Salmonella serovar when comparing the lymph node and fecal isolates (Fig. S2). Specifically, from the paired lymph node and fecal samples collected in the terminal month, 34.2% (13/38) of the paired samples shared the same Salmonella serovar. If available, these lymph node and terminal fecal pairs were matched with pen environment-manure pack isolates; identical serovars were observed in the terminal month (15.4%, 2/13) and original month (30.8%, 4/13). The relationships between these isolates can be observed in Fig. 5. Commonly, the paired lymph node and terminal fecal isolate are most closely related, but there are instances where this is not the case.

A similar finding was observed when comparing pen environment-manure pack and cattle feces collected in the terminal month; the same Salmonella serovar was identified in 15.8% (6/38) of feces collected in the terminal month and pen compared to 39.5% (15/38) from the original pen (Fig. S1). Cattle were in their original pens for a minimum of 5 months and in their new pens for a maximum of 2 months, suggesting that serovars identified within the animal itself could be related to the duration of time the cattle spent in the pen.

There were 41 pairs of pen environment-manure pack and fecal isolates originating from samples across time points other than the terminal month of the study. Of these, 41.5% (17/41) of the fecal isolates shared the same Salmonella serovar with a paired pen environment-manure pack sample. Additionally, there were 18 (with a total of 23 pairs) cattle that had 2 or more fecal isolates sequenced across the study. For these samples, 30.4% (7/23) shared the same Salmonella serovar at least once across multiple samplings (Fig. S3).

### Antimicrobial resistance.

Most of the Salmonella isolates were pansusceptible, as there were no resistance genes identified during genotypic analysis using the ResFinder database except for the single Senftenberg isolate which was multidrug resistant and included a gene encoding an extended-spectrum β-lactamase (ESBL). This isolate contained genes encoding resistance to aminoglycosides, β-lactams, sulfonamides, macrolides, tetracyclines, and phenicols. The specific resistance genes identified were: *aac(6’)-llc*, *aadA2*, *aph(3′)-la*, *aph(3″)-lb*, *aph(6)-ld*, *bla*_SHV-12_ (ESBL), *bla*_TEM-1B_, *catA2*, *ere*(A), *dfrA19*, *mcr-9*, *sul1*, *sul2*, *tet*(A), and *tet*(D). There were 134 isolates that harbored the *fosA7* gene for fosfomycin resistance, which has not been shown to encode phenotypic resistance, in our laboratory. Additionally, the aminoglycoside resistance gene *aac(6’)-Iaa* was identified in all Salmonella isolates, which was expected because it is highly conserved in Salmonella and not known to result in phenotypic resistance (38). A 16S *rrsD* rRNA point mutation for spectinomycin resistance was identified in a small group of Salmonella serovar Anatum isolates (0.76%, 4/524). There was a point mutation in the *pmrB* gene for colistin resistance in two Salmonella serovar Lubbock isolates (0.38%, 2/524). The *parC* gene, which is a quinolone resistance-determining region (QRDR), had a point mutation for an amino acid change from threonine to serine in 99.24% (521/524) of the Salmonella isolates. Strains with point mutations were not tested for phenotypic resistance or reduced susceptibility.

### Phylogenetic analysis.

**FIG 6:**
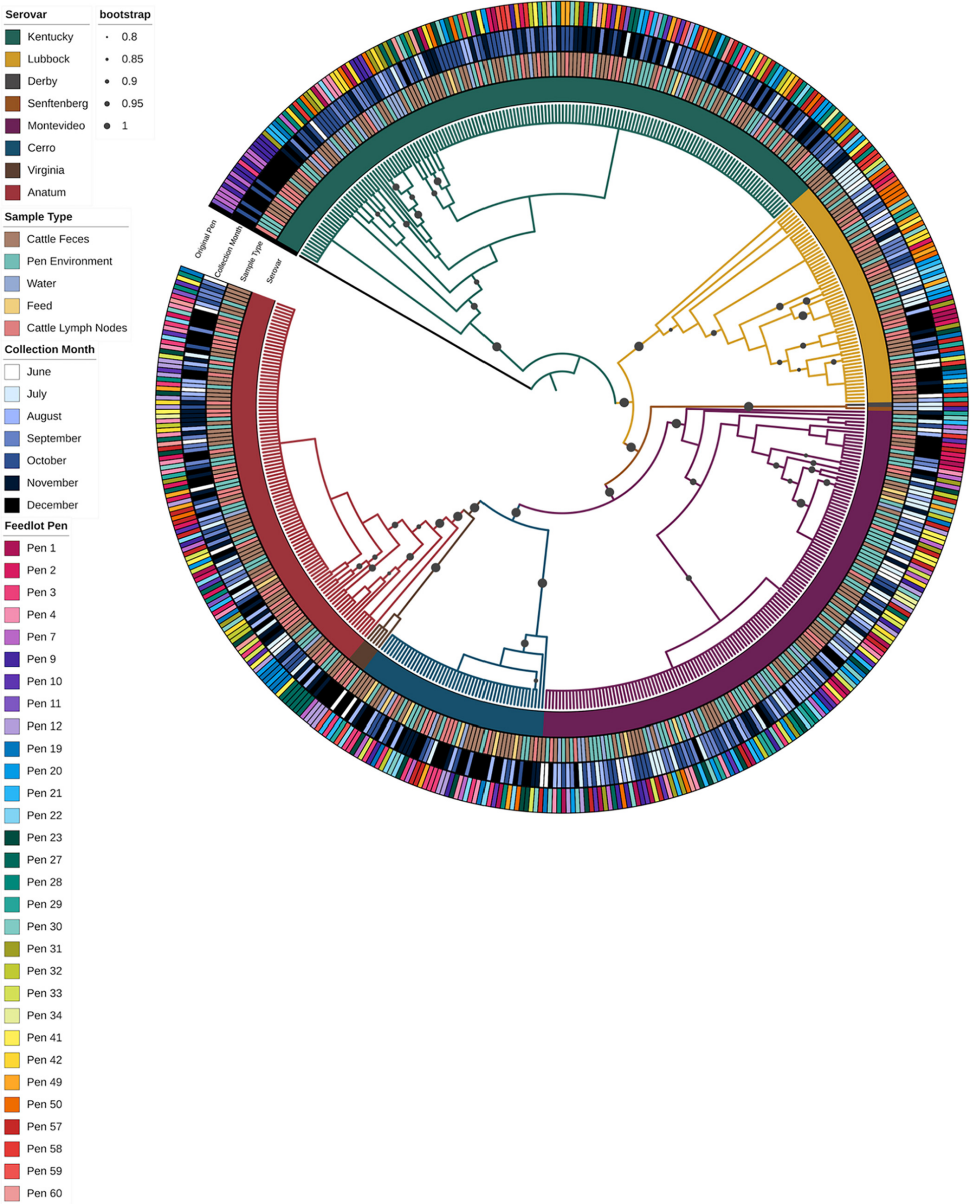
Phylogenetic tree of all sequenced Salmonella isolates arising from the longitudinal study (*n* = 524). The metadata included in the four rings surrounding the tree from the inside out are as follows: Salmonella serovar, sample type, collection month, and pen number. The pen colors are shaded by the 12 feedlot blocks. Bootstrap values are represented by the gray circles including values 0.8 to 1.0.

The complete phylogenetic tree, including all 524 isolates, shows instances where closely related isolates also show similarities across all three data types of interest (i.e., collection month, sample type, and feedlot pen). In Fig. 6, clustering by collection month was similar to previously discussed trends; Kentucky and Cerro have darker shades of blue, relating to being more prevalent in fall, Lubbock and Montevideo have lighter shades of blue, relating to being more prevalent in the late spring and early summer, and Anatum has a mix of shades of blue because it was consistently prevalent throughout the study. Sample type patterns can also be observed with most of the environmental isolates clustering in the serovar Montevideo group, feed isolates in the Cerro group, lymph node isolates in the Anatum and Lubbock groups, and fecal isolates being distributed throughout. Within sample types, lymph node and feed isolates were more closely related to fecal isolates, followed by pen environment-manure pack isolates. Water isolates were almost equally related to fecal and pen environment-manure pack isolates.

**FIG 7:**
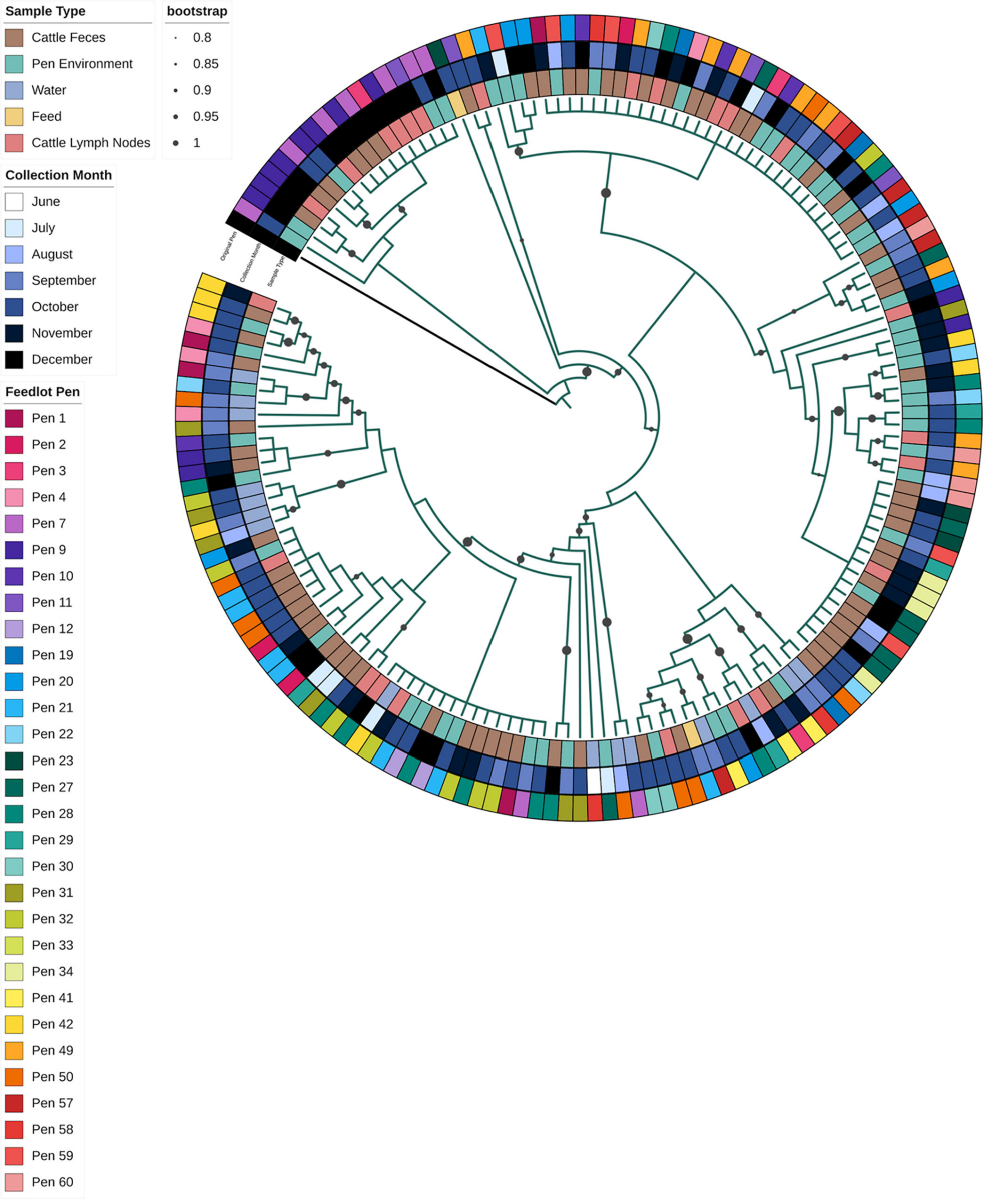
Phylogenetic tree of all Salmonella Kentucky (*n* = 162) isolates in this study. Metadata from inner ring to outer ring are as follows: original sample type, collection month, and feedlot pen, which is shaded by the 12 feedlot pen blocks. The bootstrap values are limited to values 0.8 to 1.0. The setup is the same as in Fig. 6.

Small clusters of related isolates originating from the same pen or pens within the same feedlot block were common. Within serovar Kentucky (Fig. 7), the first 18 isolates were from pens 7 to 11 (purples), except for one isolate from pen 2 (pink). A group of 16 clonal Kentucky isolates all originated from fecal samples, except for 3 isolates, and these were all from pens 23 to 34 (greens), except for 4 isolates. Other examples are a group of related water isolates that were all from pens 31 (greens) through 42 (yellow).

## DISCUSSION

A longitudinal study of beef cattle and their feedlot pen environment was conducted to observe Salmonella dynamics between environmental and host locations. We evaluated the Salmonella prevalence of 6 different sample types (pen environment-manure pack, pen environment-dry, feces, water, feed, and subiliac lymph nodes) across 7 months (June to December 2019). Differences in Salmonella serovar by sample type and season were evident. Phylogenetic analysis of related isolates confirmed associations between serovar, sample type, collection month, and feedlot pen. Antimicrobial resistance genes and point mutations were identified but were too infrequent for longitudinal analysis.

Our findings suggest that Salmonella was persistent (53.3 to 96.7%) in the feedlot environment from June to December in Texas. Salmonella prevalence in cattle environments has been shown to be highly variable and lower in other studies than in our study (76.0%, 146/192). A longitudinal study reflecting samples collected from February to October 2004 on a dairy farm in the southwestern region of the United States reported pen soil prevalence to be 39.0%; however, we were unable to compare our serovar data with that study since only the serogroup was reported (33). In a more recent study conducted from February to April of 2016, researchers randomly selected three pens from three feedlots across Texas that were previously estimated to have high, medium, or low Salmonella prevalence (36). Although the Salmonella prevalence varied considerably by feedlot, as expected (i.e., 100% [9/9], 33.3% [3/9], and 0% [0/9]), the average prevalence was 44.4% (12/27) for all soil samples (36). The serovars identified in these soil samples were mostly Anatum (64.0%, 16/25), a finding which is comparable to our study, in which Anatum was present in the environmental samples across all months. Differences in prevalence from our study could be attributed to the time of year that samples were collected, feedlot location, sample collection methods, culturing methods, or management practices.

Salmonella serovars Kentucky, Montevideo, and Cerro were more commonly identified in feedlot environmental samples, which could contribute to contamination of other pen components such as the water troughs and feed bunks. This was observed in our study, as feed and water also had high quantities of Montevideo and Cerro compared to other serovars. This is consistent with a study by Purdy et al., which identified Salmonella within feed yard water and non-feed yard water (playas) and reported that 46.7% of Salmonella isolates from water samples collected during the summer were of serovar Cerro (31). As with other sample types, Salmonella prevalence in water can be highly variable between studies. This can range from only 0.8% (2/235) of water trough samples found in the Pacific Northwest (34) to 38% of water samples collected from dairy farms in the southwestern region of the United States (33).

Identifying the origins of Salmonella in animal feed is complicated because the feed ingredients are typically mixed together and originate from several sources (39). In the previously mentioned longitudinal study by Edrington et al., total mixed rations (TMR) from feed bunks at a dairy farm were reported to have a Salmonella prevalence of 76% (33), but Dargatz et al., reported Salmonella in 5.3% of feed samples, which is similar to our reported prevalence of 8.7% (35). Our low feed sample size makes comparisons to other samples in the pen difficult; however, the majority of serovars identified were Cerro (46.2%, 6/13) and Montevideo (30.8%, 4/13), which were identified more frequently in the pen environment than in cattle samples (i.e., fecal and lymph nodes). As hypothesized by other researchers, feed and water could also become contaminated from the feedlot environment via dust particles moved by wind and cattle activity or by fecal droppings from birds and rodents (40–42). Alternatively, Salmonella-contaminated feed dust could be spread by the wind during feed delivery, contributing to the contamination of other locations in the feedlot environment. There are additional locations within and outside the feedlot that are subject to Salmonella contamination; however, the focus of this study was on dynamics between the cattle host and the feedlot pen environment.

Pen environment and cattle feces can be dispersed during cattle activity, contributing to the contamination of the pen environment and other cattle through aerosolized dust. Pen environment-dry samples were meant to represent the feces-contaminated dust spread through these activities. Cattle fecal Salmonella prevalence in our study was high throughout all months (69.8% to 87.5%) except for June (32.5%). Stephens et al. reported a 50% (25/50) prevalence from fecal grabs from the same feedlot where our study was conducted (30). In another study conducted at the West Texas A&M University (WTAMU) Research Feedlot by Levent et al., Salmonella was present in 43.7% (349/799) of fecal grab samples collected across a 6-month-long period; however, these cattle were part of a randomized trial receiving ceftiofur or tulathromycin, which may have impacted prevalence (43). Our higher prevalence (70.9%, 533/752) may be due to our samples being freshly voided feces collected from the pen floor as opposed to fecal grabs. Interestingly, Kunze et al. identified Salmonella in 30.3% (182/600) of their cattle fecal samples, which were collected from the pen floor of feedlots in the southern high plains, and the prevalence did not vary significantly across season (*P* = 0.11) (27). Their lower recovery of Salmonella could relate to the research being conducted at different feedlots; importantly, cattle received at the WTAMU Research Feedlot are predominantly considered high risk, which could contribute to Salmonella presence. Health risk cattle classifications encourage proper cattle management and assist with cattle feeding needs. High-risk cattle are those that may be stressed from recent weaning, transportation, or arrival at new environments and originate from auction markets during which they have commingled with other cattle populations (44). Kunze et al. also reported the most prevalent serovars originating from fecal samples to be Anatum (32.5%) and Montevideo (19.6%). Additionally, Salmonella was isolated in 94.1% of fecal samples collected from abattoirs in Mexico where serovar Kentucky was more likely to be recovered from feces than from other sample types (*P* = 0.02) (45). Both studies are consistent with our most frequent serovar findings of Montevideo (27.5%), Kentucky (27.5%), and Anatum (22.2%). Although Salmonella is known to exist in the pen environment at the WTAMU Research Feedlot from previous cattle cohorts, cattle feces are known to contribute to feedlot environment and cattle hide Salmonella populations.

There are several studies that have investigated Salmonella in cattle lymph nodes (20–25, 43, 45). While our study was longitudinal in nature, all lymph nodes were collected in the late fall months. However, a cross-sectional study by Gragg et al. reported a higher to lower prevalence in subiliac lymph nodes among fed cattle across summer (21.4%), fall (19.7%), and winter (1.8%) (25). This is supported by the work of Nickelson et al., who observed a significantly (*P* = 0.035) higher prevalence in subiliac lymph nodes during warmer (57.5%, 115/200) than during cooler (46.5%, 93/200) months (23). Interestingly, Nickelson et al. (21.6% Cerro, 19.7% Anatum) and Gragg et al. (44.0% Montevideo, 24.8% Anatum) reported Anatum to be the second most frequently identified serovar isolated from the subiliac lymph nodes (25). A second study by Gragg et al. also reported Anatum (27.8%) as an important serovar identified in subiliac lymph nodes, equal to serovar Kentucky (27.8%) (25, 45). Their latter findings are similar to our study, where Salmonella serovars Anatum (30.2%, 29/96), Kentucky (25.0%, 24/96), and Lubbock (17.7%, 17/96) were more commonly isolated from cattle lymph nodes than were other serovars. Our results match a previous study conducted at the WTAMU Research Feedlot reported by Levent et al. (43); their overall Salmonella prevalence in lymph nodes (85.1%, 114/134) was higher than ours, but the two most frequent serovars were Anatum (34.2%, 39/114) and Lubbock (21.9% 25/114). Salmonella in cattle can be serovar specific by region or feedlot, and identifying serovars that inhabit lymph nodes is an important factor when developing targeted Salmonella mitigation strategies for safer ground beef products. We further explored the Salmonella dynamics of cattle lymph nodes by comparing matched samples from the same animal.

Several limitations impacted our ability to analyze Salmonella dynamics matched between cattle and the feedlot environment. Random sampling supplied an unbiased picture of Salmonella present in each pen but limited our ability to compare samples (e.g., feces and lymph nodes) from the same animal. Additionally, the temporal length of this project, the quantity of samples, and the transfer of samples from different research locations resulted in a high number of missing samples. These missing samples were expected to occur at random and therefore should not introduce bias, that is, apart from the first group of lymph node samples that were not collected. The opportunity to have these lymph node samples may have provided a clearer picture of Salmonella dynamics between the environment, feces, and the cattle lymph nodes. Several observations had to be removed from the analysis as a result of normal cattle management at the WTAMU Research Feedlot. A group of cattle were moved into 6 nonstudy pens, from which there were no environmental samples collected, making it impossible to determine the origin of Salmonella from these cattle samples. This also resulted in a lack of environmental data from the terminal months to match to lymph node samples. Importantly, Salmonella contamination of the lymph nodes may have occurred at other points during the transport and slaughter process; however, this study focused on the impact of the pen environment.

Despite these challenges, relationships between fecal, lymph node, and pen environmental samples were evident. Salmonella serovars from cattle lymph node and fecal samples matched the pen environment serovar more frequently, especially if the cattle had spent a longer duration in the pen (pen duration ranged from 2 to 5 months). Serovar similarities by pen could also be observed from groups of related isolates in the phylogenetic tree. For example, serovar Lubbock isolates originated from three main pen groups in the feedlot, pens 1 to 4 (pink), pens 19 to 22 (bright blue), and pens 49 to 59 (red orange) (see Fig. S4 in the supplemental material), similarly reported by a previous study conducted at the WTAMU Research Feedlot (43). These data suggest that the pen environment was more similar to feces than to lymph nodes; paradoxically, cattle lymph nodes were more similar to feces than to the environment. The ability of different Salmonella serovars (i.e., Montevideo or Anatum) to survive in different environments (i.e., environment or lymph nodes) may also contribute to this trend. We did not collect cattle hide samples during this study, which might have been expected to be more related to the Salmonella identified in the pen environment-dry samples because of aerosolized dust or to the cattle lymph nodes since transdermal transmission via insect bites has been hypothesized and experimentally reproduced (46).

The lack of AMR in the Salmonella isolates made it difficult to assess AMR associations involving the feedlot environment and cattle hosts. We did expect to identify more resistant isolates, since previous studies conducted at the WTAMU Research Feedlot have reported AMR Salmonella at rates of 38.4% and 20.1%, respectively, with tetracycline resistance being most common (43, 47). However, in these previous studies cattle were administered therapeutic doses of antibiotics as part of the experimental design, creating selective pressures. Interestingly, most of our isolates had a point mutation in the *parC* gene. This is part of the QRDR, in which mutations can result in quinolone resistance (or reduced susceptibility). It has been suggested that the *parC* gene requires a double mutation, or else must be complemented with another mutation from a different gene in the QRDR, to result in high-level phenotypic resistance (48). Antimicrobial treatments were not a part of the experimental design of the study; however, cattle were previously enrolled in a study investigating the efficacy of metaphylactic antibiotics and vaccinations for bovine respiratory disease (49). Without the administration of experimental antibiotic treatments, selective pressures were likely not optimal to select or maintain AMR Salmonella within the cattle or feedlot environment.

This study presents a longitudinal comparison of Salmonella in the feedlot environment (including two types of environmental samples, water, and feed) and a single cohort of cattle (freshly voided feces and pooled subiliac lymph nodes). Salmonella prevalence varied significantly by collection month for most sample types. There was a difference in Salmonella prevalence for cattle feces and pen environment based on diets containing SB or WDGS. Collection month and sample type impacted which Salmonella serovars were identified the majority of the time. The serovars identified in this study are not typically associated with human salmonellosis, and the AMR Salmonella were not a significant concern. However; based on the observed Salmonella dynamics occurring between the feedlot environment and cattle host, developing Salmonella mitigation techniques with consideration given to Salmonella serovars is likely to be important. Serovars Anatum, Kentucky, and Lubbock were most frequently identified in the subiliac lymph nodes, which has also been observed in similar research at other Texas feedlots, making these serovars important targets for reducing ground beef contamination in this region (23, 43). These serovars were also identified in the feedlot environment at high prevalence, along with Montevideo. Preharvest interventions applied to the feedlot environment are a potential mitigation strategy to target specific Salmonella serovars identified in both the environment and cattle lymph nodes.

## MATERIALS AND METHODS

### Ethics statement.

This study was approved, to be conducted at the West Texas A&M University Research Feedlot, by the West Texas A&M University/Cooperative Research, Educational and Extension Team (WT/CREET) Institutional Animal Care and Use Committee (IACUC) under permit no. 2021.05.001 entitled “Harnessing the ecological dynamics of naturally occurring bacteriophage in the feedlot environment to control multidrug-resistant Salmonella in slaughter-ready beef cattle.” Microbiological research was approved by the Texas A&M University Institutional Biosafety Committee (IBC) and was conducted under IBC permits no. 2017-021 and no. 2020-069.

### Feedlot and nutritional study design.

**FIG 8:**
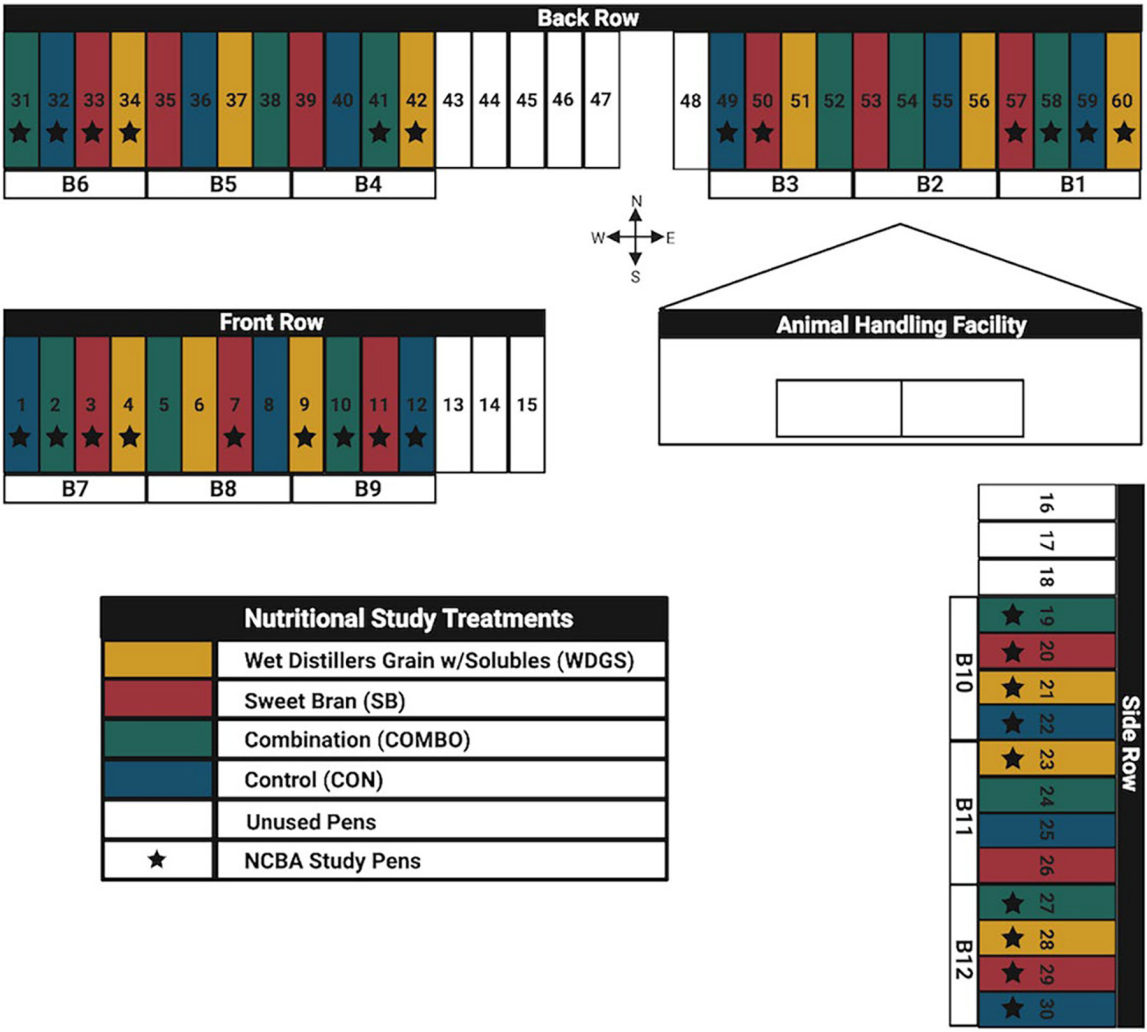
The geographical layout (3 rows [i.e., front, back, and side], 12 blocks, and 60 pens) of the West Texas A&M University Research Feedlot. Dietary treatments (yellow, WDGS; red, SB; green, COMBO; blue, CON; white, unused) were provided to cattle at the pen level. Dietary treatments and geographic locations were evenly distributed across the 30 pens selected for the longitudinal study (black stars). NCBA, National Cattlemen’s Beef Association.

Longitudinal sample collection for this study occurred at the West Texas A&M University (WTAMU) Research Feedlot in Canyon, TX, across 7 months from June to December 2019. The research feedlot consists of 60 pens (27.4 m by 6.10 m) divided into 12 blocks across 3 rows (Fig. 8). An ongoing nutritional study, previously described by Spowart et al., used 48 pens at the feedlot to house 9 to 10 beef cattle (*n* = 478) in each pen (50). Cattle placement was based on body weight and the number of previous antibiotic treatments received. The nutritional study consisted of four dietary treatments applied at the pen level, which included steam-flaked corn-based finishing diets containing wet distillers’ grains with solubles (WDGS), Sweet Bran (SB; Cargill Branded Feed, Minneapolis, MN), a combination of WDGS and SB (COMBO), and a control (CON) treatment in which cattle did not receive any corn-milling products in the diet. Samples for our longitudinal study were collected from 30 of the feedlot pens, including 282 beef cattle, which were evenly distributed across the four dietary treatment groups and geographic locations within the feedlot.

### Sampling days.

**FIG 9:**
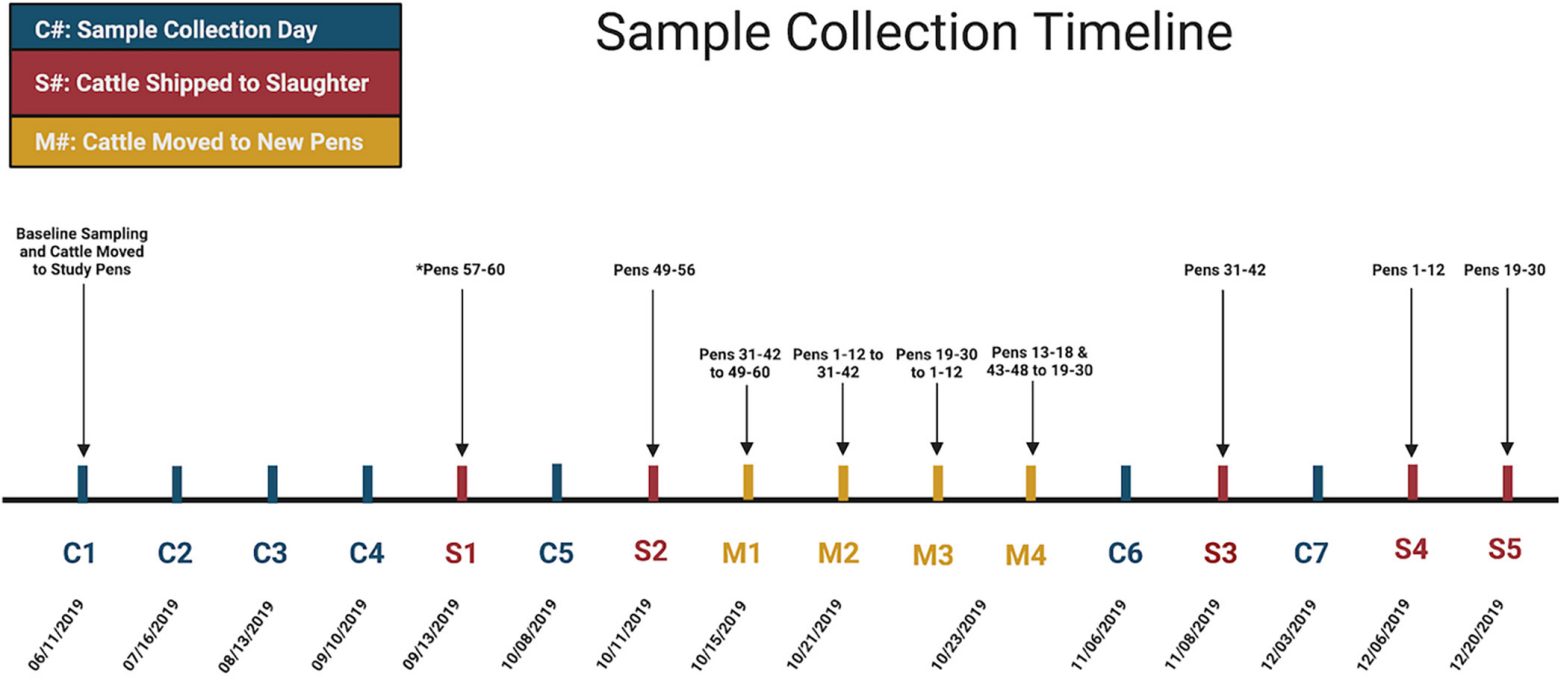
Sample collection timeline of the longitudinal study which consisted of three main types of events. Sample collections are signified by blue markers, cattle slaughter dates by red markers, and movements of cattle between pens by yellow markers.

A baseline sampling of cattle feces and pen environment was collected before cattle relocation from the nutritional study pens into the longitudinal study pens (Fig. 9). Water troughs were cleaned each Wednesday at the research feedlot by scrubbing the trough with a nylon brush, draining the water, and refilling. Therefore, all sample types were collected on Tuesdays when pen water troughs were considered their dirtiest. Collections occurred every 28 days, except for the second collection, which occurred 35 days after the previous collection.

### Sample collection.

Six different sample types were collected: freshly voided cattle feces, pen environment-manure pack, pen environment-dry, pen feed, pen water, and bovine left and right subiliac lymph nodes (once, at slaughter only). A monthly sample per pen (*n* = 30) was collected for all environmental, feed, and water samples except for November and December, when feed and water samples were not collected because of low Salmonella prevalence. Freshly voided fecal samples were collected from four randomly selected cattle per pen each month (*n* = 120). As cattle went to slaughter starting in October 2019, described in detail below, the number of samples collected each month declined (see Table S1 in the supplemental material).

Approximately 25 g of solid sample or 25 mL of liquid sample was collected for each sample type. Freshly voided feces were collected from the pen floor using a disposable plastic spoon. Pen environment-manure pack samples were composites collected from 6 locations across a transect of each pen surface, and pen environment-dry samples were composites collected from 6 locations along the 2 outside edges of the pen meant to represent the desiccated pen environment that could become aerosolized. Water and feed samples were taken directly from the feed bunk or water trough. Samples were collected in the mornings and shipped overnight from the WTAMU Research Feedlot to Texas A&M University (TAMU), School of Veterinary Medicine & Biomedical Sciences in College Station, TX, for processing.

Once cattle reached market weight, they were shipped by pen blocks for slaughter. Left and right subiliac lymph nodes from each animal were harvested prechill at the slaughter plant. The left and right lymph nodes (*n* = 454) for each carcass were pooled (*n* = 227) as one sample and transported to the Food Microbiology Laboratory in the TAMU Department of Animal Science in College Station, TX, for processing.

As cattle were shipped for slaughter, pen water troughs became available for repair, and the remaining cattle were shifted in groups to different pen blocks. The pens that cattle were moved from would subsequently be repaired, allowing for the next group of cattle to be moved until all cattle were moved into different pens prior to being sent to slaughter (Table S2).

### Sample processing.

For selective enrichment culturing methods of Salmonella, 0.5 g of each solid sample (feces, pen environment-manure pack, and pen environment-dry) or 0.5 mL of liquid sample (water) was added to 5 mL tryptic soy broth (TSB; Becton, Dickinson and Company [BD], Franklin Lakes, NJ, USA) and incubated for 3 h at 42°C. A 1-mL aliquot of the TSB culture was transferred into 9 mL of tetrathionate broth (TTB; BD, Franklin Lakes, NJ, USA) supplemented with 200 μL of iodine (BD, Franklin Lakes, NJ, USA) and incubated for 24 h at 37°C. The following day, 100 μL of the TTB culture was transferred to 10 mL of Rappaport-Vassiliadis broth 10 (RV; BD, Franklin Lakes, NJ, USA) and incubated for 19 h at 42°C. An Eddy Jet 2 spiral plater (Neutec Group Inc., Farmingdale, NY, USA) was used, for ease of bacterial colony isolation, to plate 50 μL of each RV culture onto brilliant green agar (BGA; BD, Franklin Lakes, NJ, USA) plates supplemented with 25 mg/L novobiocin (Sigma-Aldrich, St. Louis, MO, USA) to selectively grow Salmonella during an incubation of 19 h at 37°C. Two phenotypic Salmonella-like appearing colonies per positive BGA plate, as indicated by bright pink agar and whitish pink colonies, were streaked onto tryptic soy agar with 5% sheep’s blood agar plates (BD, Franklin Lakes, NJ, USA) and incubated for 19 to 24 h at 37°C. Two white, nonhemolytic isolates from each blood agar plate were streaked in duplicates onto a target plate for matrix-assisted laser desorption ionization–time of flight mass spectrometry (MALDI-TOF MS; Bruker, Billerica, MA, USA) to confirm bacterial isolates as Salmonella. All Salmonella confirmed isolates were saved using the CryoCare bead storage system (cryobeads; Scientific Device Laboratory, Des Plaines, IL, USA) and stored at −80°C for future use.

For feed samples, 0.5 g of the sample was placed in a Stomacher 400 circulator strainer bag (Seward, Worthington, UK) with 9 mL of TSB and loaded into a Stomacher 400 circulator (Seward, Worthington, UK) for 2 min at 230 rpm to simulate mechanical digestion before continuing with the previously mentioned selective enrichment protocol. The subiliac lymph nodes from each animal were dipped in ethanol and flame sterilized, excess fatty tissue was removed, and the lymph nodes were flame sterilized again. Lymph nodes were placed into a filter bag (Seward, Worthington, UK), weighed, and pulverized with a rubber mallet. Modified tryptone soya broth (mTSB) (Oxoid Ltd., Basingstoke, Hampshire, United Kingdom) supplemented with 20 mg/L novobiocin (Oxoid Ltd., Basingstoke, Hampshire, United Kingdom) was added to the sample bag for a 1:4 ratio using the combined weight of the pooled lymph nodes from each carcass. The samples were incubated for 15 h at 37°C and removed for standardized BAX system PCR assay (Hygiena, Camarillo, CA, USA) screening. Only the presumptive Salmonella-positive cultures (based on the BAX results) underwent the entirety of the selective enrichment process as described above. BAX screening was used only for lymph node samples, as this method was developed for food safety labs to detect Salmonella in samples destined for food products in postharvest settings. It has not been validated for fecal samples or environmental samples (other than environmental swabs) in the preharvest setting.

### DNA extraction.

All Salmonella confirmed isolates from pen environment-manure pack (*n* = 145), water (*n* = 34), feed (*n* = 13), and lymph node (*n* = 96) samples underwent DNA extraction. If available, one Salmonella confirmed isolate originating from fecal (*n* = 171) samples from each pen per month was randomly selected for DNA extraction. Salmonella DNA was extracted from an additional 35 isolates from a secondary sampling in October and also from 30 isolates originating in cattle fecal samples collected during the terminal month that were paired with lymph node isolates from the same animal. Preparation involved streaking a 1-μL loop from the isolate cryobeads to a blood agar plate with an overnight incubation at 37°C followed by the transfer of a single colony into 4 mL of TSB for overnight incubation at 37°C. Standard procedures for the Qiagen QIAamp 96 DNA isolation kit with a QIAcube HT instrument (Qiagen, Hilden, Germany) were followed to extract DNA from the overnight cultures, as previously described (47). Upon completion, 2 μL of DNA was measured for quantity and quality on a LVis plate with a FLUOstar Omega multiplate reader (BMG Labtech, Ortenberg, Germany) using MARS data analysis software. If the 260-/280-nm ratio was outside the 1.75 to 2.05 range, DNA was reextracted from those isolates. DNA was stored at −30°C prior to use for sequencing.

### Whole-genome sequencing.

Before library preparation, double-stranded DNA concentrations (nanograms per microliter) were measured using a Qubit double-stranded DNA (dsDNA) high-sensitivity (HS) assay kit (Thermo Fisher Scientific, Waltham, MA, USA) with a Qubit 4 fluorometer (Thermo Fisher Scientific, Waltham, MA, USA) to calculate the amount (1 to 17 μL) of eluted DNA per sample for initial DNA library preparation steps. Individual DNA libraries were prepared using Swift 2S Turbo library preparation kits (Swift Biosciences, Ann Arbor, MI, USA), 32 to 36 isolates at a time, per standard procedure. Briefly, DNA was enzymatically fragmented prior to adapter ligation followed by unique PCR indexing and amplification. To determine library quality, the prepared library concentration (nanograms per microliter) was measured with a Qubit 4 Fluorometer as previously described, and fragment size (base pairs) was determined with an NGS standard sensitivity fragment analysis kit (1 to 6,000 bp) (Agilent Technologies, Santa Clara, CA, USA) using a Fragment Analyzer automated capillary electrophoresis (CE) system (Advanced Analytical, Des Moines, IA, USA). A final DNA library concentration of 12 nM or higher was considered acceptable for sequencing. Libraries were then subjected to the Swift Normalase kit (Swift Biosciences, Ann Arbor, MI, USA) to account for individual library variation and to accelerate library pooling. Library denaturation and dilution were conducted according to the standard protocol for an Illumina MiSeq V2 500-cycle cartridge. Pooled libraries were loaded at a 12 pM concentration into the cartridge and run on an Illumina MiSeq platform (Illumina, San Diego, CA, USA) for 250 paired-end cycles.

### Bioinformatics.

The High-Performance Research Computing (HPRC) system at TAMU was used to run batches of sequenced reads through a bioinformatics pipeline. Bioinfokit v.0.7.2 was used as a quick method to determine coverage depth prior to complete read analysis, ensuring that any samples with coverage depths below 30× underwent new library preparation and resequencing (51). To assess the quality of the reads, FastQC v.0.11.9 results were parsed by MultiQC v.1.7 to generate a report of relevant information, including genome size, GC content, *N*_50_ (base pairs), and largest contig (base pairs) (52, 53). Trimmomatic v.0.39 was used to trim off adapters from the reads prior to assembly (54). St. Petersburg genome assembler (SPAdes) v.3.14.0 was used for the assembly of Illumina paired-end reads, which then were assessed with the Quality Assessment Tool (QUAST) v.5.0.2 (55, 56). SeqSero v.1.0.1 identified genes corresponding to antigens to predict the antigenic formula for each sequenced isolate for serotyping Salmonella (57). Multilocus sequence typing (MLST) v.2.1.17 determined the legacy sequence type (ST) using the 7-locus genes for Salmonella (58). ResFinder through the Abricate v.0.9.9 platform was used to determine the presence of AMR genes (59). ResFinder searched for the presence of aminoglycoside, amphenicol, beta-lactamase, fosfomycin, macrolide, quinolone, sulfonamide, tetracycline, and multidrug transporter resistance genes. PointFinder was used to identify point mutations for resistance genes by first comparing to a chromosomal gene database and then comparing mismatches to a point mutation database (60). Assemblies were run through Parsnp v.1.2 for comparison to a compressed suffix graph index from the complete reference genome, and final single nucleotide polymorphisms (SNPs) were used to create maximum likelihood phylogenies through FastTree2 (61, 62). Parsnp combines core genome alignment and read mapping in order to account for structural, point, and indel mutations. Our phylogenetic analysis included the original 459 isolates, the 30 paired isolates as previously discussed, and an additional 35 isolates from a second and third sampling in October. The complete reference genome of a Salmonella Kentucky isolate from NCBI (GenBank accession no. CP082582) was used for the full phylogenetic tree alignment. Alignments of the full phylogenetic tree using complete reference genomes of the other serovars were conducted, and no differences were observed as isolates always cluster by serovar; therefore, we selected serovar Kentucky, which was frequently observed, for our reference genome. Individual serovar phylogenetic trees were constructed using complete reference genomes of each serovar (GenBank accession numbers: Anatum, CP074259; Cerro, CP082902; Kentucky, CP082582; Lubbock, CP032814; and Montevideo, CP082436) for higher resolution and can be found in Fig. S4. The trees and metadata were uploaded into iTOL v.6.5.7 to create phylogenetic graphics with improved visualization (63). Color-coded metadata, including serovar, sample type, collection month, and feedlot pen, were added to the phylogenetic trees using the iTOL annotation editor to visualize and quantify associations between related Salmonella isolates.

### Statistical analysis.

Comparative statistics were completed using Stata/IC version v.17.0 (StataCorp, College Station, TX, USA), and RStudio v.1.4.1717 was used to develop prevalence and serovar graphics (64). For all statistics, an alpha value of 0.05 or lower was considered statistically significant. A multilevel mixed-effects logistic regression model was created to evaluate Salmonella prevalence by individual sample type. The fixed indicator variables were collection month and dietary treatment; specifically, dietary treatment was included as a forced interaction term to account for the 2 × 2 full-factorial design. Feedlot pen was a random effect variable accounting for clustering within feedlot pens. Predictive margins for Salmonella prevalence were determined for all sample types. Similarly, a multinomial logistic regression model with sample type, dietary treatment, and collection month as fixed variables and pen as a random effects panel variable with an independent variance-covariance structure was developed to evaluate relationships for Salmonella serovars. Serovar Montevideo was set as the base serovar for all comparisons because it was most commonly identified. Fixed effect base levels included feces for sample type because it was the most abundant, control for dietary treatment, and June for collection month because it was the baseline collection. Serovars Virginia, Derby, and Senftenberg violated the minimum cell value requirements and were originally collapsed into an “Other” category, but these serovars were ultimately excluded to permit the multinomial model to converge. A multiple correspondence analysis constructed with Burt’s matrices and principal normalization was used to visualize the relationships between serovar, fixed effect variables, and random effect variables. The same approach was used to visualize serovar trends by feedlot pen. BioRender was used to create infographics (BioRender, Toronto, ON, Canada).

### Data availability.

Sequencing data for this project can be found under NCBI BioProject accession number PRJNA807300.
