# Supplementary material for: A Longitudinal Study on the Dynamics of Salmonella enterica Prevalence and Serovar Composition in Beef Cattle Feces and Lymph Nodes and Potential Contributing Sources from the Feedlot Environment
Source: Appl Environ Microbiol. 2023 Apr 6;89(4):e00033-23. doi: 10.1128/aem.00033-23 (PMC10132121; doi:10.1128/aem.00033-23)
Supplement: Supplemental file 1 — Supplemental material. Download aem.00033-23-s0001.docx, DOCX file, 2.4 MB [file aem.00033-23-s0001.docx]

**SUPPLEMENTAL MATERIAL**

| **Table S1.** Number of samples collected and prevalence by month and sample type. | | | | | | | | |
| --- | --- | --- | --- | --- | --- | --- | --- | --- |
| Sample Type | *Month of Sample Collection* | | | | | | | |
|  | *June* | *July* | *Aug* | *Sept* | *Oct* | *Nov* | *Dec* | *Total* |
| Fecal | 0.325(39/120) | 0.750(90/120) | 0.750(90/120) | 0.875(105/120) | 0.788(82/104) | 0.698(67/96) | 0.833(60/72) | 0.709(533/752) |
| Comp. Envi | 0.533(16/30) | 0.733(22/30) | 0.633(19/30) | 0.967(29/30) | 0.800(24/30) | 0.792(19/24) | 0.944(17/18) | 0.760(146/192) |
| Comp. Envi. Dry | 0.367(11/30) | 0.633(19/30) | 0.800(24/30) | 0.700(21/30) | 0.885(23/26) | 0.792(19/24) | 0.778(14/18) | 0.697(131/188) |
| Water | 0.033(1/30) | 0.267(8/30) | 0.200(6/30) | 0.400(12/30) | 0.308(8/26) | N/A^*^(0/0) | N/A (0/0) | 0.240(35/146) |
| Feed | 0.000(0/30) | 0.033(1/30) | 0.267(8/30) | 0.067(2/30) | 0.069(2/29) | N/A (0/0) | N/A (0/0) | 0.087(13/149) |
| Lymph Nodes | N/A (0/0) | N/A (0/0) | N/A (0/0) | N/A (0/0) | 1.000(17/17) | 0.373(22/59) | 0.377(57/151) | 0.423(96/227) |
| Total | 0.279(67/240) | 0.583(140/240) | 0.613(147/240) | 0.704(169/240) | 0.672(156/232) | 0.626(127/203) | 0.571(148/259) | 0.577(954/1,654) |

* N/A - samples not collected

| ***Table S2***. Cattle and treatment allocations at WTAMU Research Feedlot including pen movement. | | | | | | |
| --- | --- | --- | --- | --- | --- | --- |
| *Dietary* Treatment* | *Number of Cattle* | *Original Pen Number* | | *Cattle Move #1* | *Cattle Move #2* | *Cattle Move #3* |
| CON | 9 | | 1 | 1 | 31 | 31 |
| COMBO | 9 | | 2 | 2 | 32 | 32 |
| *SB^®^* | 9 | | 3 | 3 | 33 | 33 |
| WDGS | 9 | | 4 | 4 | 34 | 34 |
| *SB^®^* | 9 | | 7 | 7 | 37 | 37 |
| WDGS | 9 | | 9 | 9 | 39 | 39 |
| COMBO | 9 | | 10 | 10 | 40 | 40 |
| *SB^®^* | 9 | | 11 | 11 | 41 | 41 |
| CON | 9 | | 12 | 12 | 42 | 42 |
| COMBO | 9 | | 19 | 19 | 19 | 1 |
| *SB^®^* | 9 | | 20 | 20 | 20 | 2 |
| WDGS | 9 | | 21 | 21 | 21 | 3 |
| CON | 9 | | 22 | 22 | 22 | 4 |
| WDGS | 9 | | 23 | 23 | 23 | 7 |
| COMBO | 9 | | 27 | 27 | 27 | 9 |
| WDGS | 9 | | 28 | 28 | 28 | 10 |
| *SB^®^* | 9 | | 29 | 29 | 29 | 11 |
| CON | 9 | | 30 | 30 | 30 | 12 |
| COMBO | 10 | | 31 | 49 | 49 | 49 |
| CON | 10 | | 32 | 50 | 50 | 50 |
| *SB^®^* | 10 | | 33 | 51 | 51 | 51 |
| WDGS | 10 | | 34 | 52 | 52 | 52 |
| COMBO | 10 | | 41 | 59 | 59 | 59 |
| WDGS | 10 | | 42 | 60 | 60 | 60 |
| CON | 10 | | 49 | N/A** | N/A | N/A |
| *SB^®^* | 10 | | 50 | N/A | N/A | N/A |
| *SB^®^* | 10 | | 57 | N/A | N/A | N/A |
| COMBO | 10 | | 58 | N/A | N/A | N/A |
| CON | 10 | | 59 | N/A | N/A | N/A |
| WDGS | 10 | | 60 | N/A | N/A | N/A |
|  | 282 Cattle | | 30 Pens |  |  |  |

*WDGS = wet distillers’ grain with solubles, *SB^®^* = *Sweet Bran^®^,* COMBO = both WDGS and *SB^®^*, CON = control, no supplementation

** N/A - samples not collected

| **Table S3**. Values from the multilevel mixed effects logistic regression model for Salmonella  prevalence including standard errors, p-values and 95% confidence intervals. | | | | | | |
| --- | --- | --- | --- | --- | --- | --- |
|  |  | *Coefficient* | *SE* | *p-value* | *95% Confidence Intervals* | |
| Covariate | *Fecal Samples* | | | | | |
| Collection Month | *July* | 1.933 | 0.298 | 0.000 | 1.350 | 2.517 |
|  | *August* | 1.933 | 0.298 | 0.000 | 1.350 | 2.517 |
|  | *September* | 2.811 | 0.349 | 0.000 | 2.127 | 3.496 |
|  | *October* | 2.183 | 0.323 | 0.000 | 1.550 | 2.817 |
|  | *November* | 1.672 | 0.310 | 0.000 | 1.066 | 2.279 |
|  | *December* | 2.447 | 0.386 | 0.000 | 1.691 | 3.204 |
| Dietary Treatment | *WDGS* | 0.751 | 0.337 | 0.026 | 0.091 | 1.411 |
|  | *SB* | 0.082 | 0.325 | 0.801 | -0.555 | 0.719 |
|  | *COMBO* | -0.473 | 0.471 | 0.315 | -1.396 | 0.450 |
|  | *Composite Environment Fecal Pack Samples* | | | | | |
| Collection Month | *July* | 0.962 | 0.582 | 0.098 | -0.178 | 2.102 |
|  | *August* | 0.454 | 0.553 | 0.412 | -0.631 | 1.538 |
|  | *September* | 3.427 | 1.110 | 0.002 | 1.251 | 5.604 |
|  | *October* | 1.366 | 0.618 | 0.027 | 0.156 | 2.577 |
|  | *November* | 1.299 | 0.655 | 0.047 | 0.015 | 2.583 |
|  | *December* | 2.887 | 1.125 | 0.010 | 0.683 | 5.091 |
| Dietary Treatment | *WDGS* | -0.132 | 0.613 | 0.829 | -1.334 | 1.069 |
|  | *SB* | -1.360 | 0.584 | 0.020 | -2.504 | -0.216 |
|  | *COMBO* | 0.871 | 0.812 | 0.284 | -0.721 | 2.462 |
|  | *Composite Environment Dry Samples* | | | | | |
| Collection Month | *July* | 1.198 | 0.567 | 0.035 | 0.087 | 2.309 |
|  | *August* | 2.106 | 0.633 | 0.001 | 0.866 | 3.346 |
|  | *September* | 1.525 | 0.584 | 0.009 | 0.381 | 2.670 |
|  | *October* | 2.808 | 0.768 | 0.000 | 1.303 | 4.312 |
|  | *November* | 2.073 | 0.677 | 0.002 | 0.746 | 3.401 |
|  | *December* | 2.017 | 0.737 | 0.006 | 0.573 | 3.462 |
| Dietary Treatment | *WDGS* | 0.345 | 0.606 | 0.570 | -0.843 | 1.532 |
|  | *SB* | -0.341 | 0.585 | 0.560 | -1.488 | 0.807 |
|  | *COMBO* | -0.146 | 0.838 | 0.862 | -1.788 | 1.496 |
|  | *Water Trough Samples* | | | | | |
| Collection Month | *July* | 2.407 | 1.105 | 0.029 | 0.242 | 4.572 |
|  | *August* | 2.016 | 1.121 | 0.072 | -0.181 | 4.213 |
|  | *September* | 3.053 | 1.092 | 0.005 | 0.912 | 5.193 |
|  | *October* | 2.616 | 1.110 | 0.018 | 0.440 | 4.792 |
| Dietary Treatment | *WDGS* | 1.350 | 0.727 | 0.063 | -0.074 | 2.774 |
|  | *SB* | 1.633 | 0.718 | 0.023 | 0.225 | 3.041 |
|  | *COMBO* | -1.421 | 0.907 | 0.117 | -3.198 | 0.357 |
|  | *Feed Samples* | | | | | |
| Collection Month | *July* | -0.908 | 1.299 | 0.485 | -3.454 | 1.638 |
|  | *August* | 1.844 | 0.926 | 0.047 | 0.029 | 3.660 |
|  | *September* | -0.126 | 1.090 | 0.908 | -2.263 | 2.010 |
|  | *October* | - | - | - | - | - |
| Dietary Treatment | *WDGS* | -1.664 | 0.821 | 0.043 | -3.273 | -0.056 |
|  | *SB* | -2.914 | 1.166 | 0.012 | -5.198 | -0.629 |
|  | *COMBO* | 1.809 | 1.683 | 0.282 | -1.490 | 5.107 |
|  | *Lymph Node Samples* | | | | | |
| Collection Month | *October* | - | - | - | - | - |
|  | *November* | 0.006 | 0.324 | 0.985 | -0.628 | 0.641 |
|  | *December* | - | - | - | - | - |
| Dietary Treatment | *WDGS* | -0.170 | 0.425 | 0.689 | -1.002 | 0.662 |
|  | *SB* | 0.392 | 0.425 | 0.355 | -0.440 | 1.225 |
|  | *COMBO* | 0.026 | 0.578 | 0.964 | -1.107 | 1.158 |

| **Table S4**. Values from the multinomial logistic regression model for Salmonella serovars including standard errors, p-values and 95% confidence intervals. | | | | | | |
| --- | --- | --- | --- | --- | --- | --- |
|  |  | *Coefficient* | *SE* | *p-value* | *95% Confidence Intervals* | |
| Covariate | *Serovar Anatum* | | | | | |
| Sample Type | *Environment* | -0.735 | 0.342 | 0.032 | -1.406 | -0.064 |
|  | *Water* | -1.644 | 0.821 | 0.045 | -3.253 | -0.035 |
|  | *Feed* | -1.124 | 1.175 | 0.339 | -3.426 | 1.725 |
|  | *Lymph Nodes* | 0.700 | 0.523 | 0.180 | -0.324 | 1.725 |
| Collection Month | *July* | -0.677 | 0.631 | 0.283 | -1.914 | 0.560 |
|  | *August* | -0.405 | 0.618 | 0.512 | -1.615 | 0.806 |
|  | *September* | 0.090 | 0.605 | 0.882 | -1.095 | 1.275 |
|  | *October* | -0.846 | 0.686 | 0.218 | -2.192 | 0.499 |
|  | *November* | 0.287 | 0.671 | 0.669 | -1.029 | 1.603 |
|  | *December* | -0.097 | 0.709 | 0.891 | -1.487 | 1.293 |
| Dietary Treatment | *WDGS* | 0.434 | 0.439 | 0.323 | -0.426 | 1.294 |
|  | *SB* | -0.327 | 0.458 | 0.475 | -1.224 | 0.570 |
|  | *COMBO* | 0.694 | 0.437 | 0.113 | -0.163 | 1.551 |
|  | *Serovar Cerro* | | | | | |
| Sample Type | *Environment* | -0.702 | 0.531 | 0.187 | -1.743 | 0.340 |
|  | *Water* | 1.585 | 0.808 | 0.050 | 0.002 | 3.168 |
|  | *Feed* | 2.541 | 0.901 | 0.005 | 0.774 | 4.307 |
|  | *Lymph Nodes* | -1.349 | 0.673 | 0.045 | -2.669 | -0.029 |
| Collection Month | *July* | -1.478 | 1.523 | 0.332 | -4.464 | 1.507 |
|  | *August* | 0.181 | 1.211 | 0.881 | -2.192 | 2.554 |
|  | *September* | -0.674 | 1.351 | 0.681 | -3.322 | 1.974 |
|  | *October* | 0.816 | 1.197 | 0.496 | -1.531 | 3.162 |
|  | *November* | 2.350 | 1.188 | 0.048 | 0.021 | 4.679 |
|  | *December* | 3.065 | 1.175 | 0.009 | 0.761 | 5.369 |
| Dietary Treatment | *WDGS* | -0.275 | 0.659 | 0.676 | -1.567 | 1.016 |
|  | *SB* | -0.033 | 0.628 | 0.958 | -1.265 | 1.198 |
|  | *COMBO* | -2.839 | 1.175 | 0.016 | -5.142 | -0.536 |
|  | *Serovar Kentucky* | | | | | |
| Sample Type | *Environment* | -0.431 | 0.339 | 0.204 | -1.095 | 0.234 |
|  | *Water* | 0.717 | 0.546 | 0.189 | -0.354 | 1.788 |
|  | *Feed* | -0.091 | 1.029 | 0.930 | -2.107 | 1.925 |
|  | *Lymph Nodes* | -0.938 | 0.514 | 0.068 | -1.946 | 0.070 |
| Collection Month | *July* | 0.926 | 1.212 | 0.445 | -1.450 | 3.302 |
|  | *August* | 1.342 | 1.190 | 0.259 | -0.990 | 3.675 |
|  | *September* | 2.812 | 1.160 | 0.015 | 0.539 | 5.086 |
|  | *October* | 3.686 | 1.176 | 0.002 | 1.382 | 5.991 |
|  | *November* | 4.106 | 1.220 | 0.001 | 1.715 | 6.496 |
|  | *December* | 4.275 | 1.242 | 0.001 | 1.840 | 6.710 |
| Dietary Treatment | *WDGS* | 0.424 | 0.697 | 0.543 | -0.942 | 1.790 |
|  | *SB* | -0.328 | 0.704 | 0.641 | -1.708 | 1.052 |
|  | *COMBO* | -0.316 | 0.732 | 0.666 | -1.752 | 1.119 |
|  | *Serovar Lubbock* | | | | | |
| Sample Type | *Environment* | -0.616 | 0.418 | 0.141 | -1.435 | 0.203 |
|  | **Water* | -24.397 | 55837.24 | 1.000 | -109463.4 | 109414.6 |
|  | **Feed* | -29.896 | 2260537 | 1.000 | -4430600 | 4430540 |
|  | *Lymph Nodes* | 1.622 | 0.741 | 0.029 | 0.169 | 3.075 |
| Collection Month | *July* | -0.739 | 0.619 | 0.232 | -1.951 | 0.474 |
|  | *August* | -1.495 | 0.701 | 0.033 | -2.869 | -0.120 |
|  | *September* | -1.178 | 0.678 | 0.082 | -2.506 | 0.151 |
|  | *October* | -2.334 | 0.837 | 0.005 | -3.974 | -0.694 |
|  | *November* | -2.192 | 0.925 | 0.018 | -4.005 | -0.379 |
|  | *December* | -1.962 | 0.918 | 0.032 | -3.761 | -1.164 |
| Dietary Treatment | *WDGS* | -0.710 | 0.788 | 0.367 | -2.253 | 0.834 |
|  | *SB* | -0.128 | 0.725 | 0.860 | -1.549 | 1.294 |
|  | *COMBO* | 0.415 | 0.746 | 0.578 | -1.048 | 1.877 |
|  | *Serovar Montevideo-Base Outcome* | | | | | |

*Important to note that the model is highly unstable for *Salmonella* serovar Lubbock in water and feed samples.


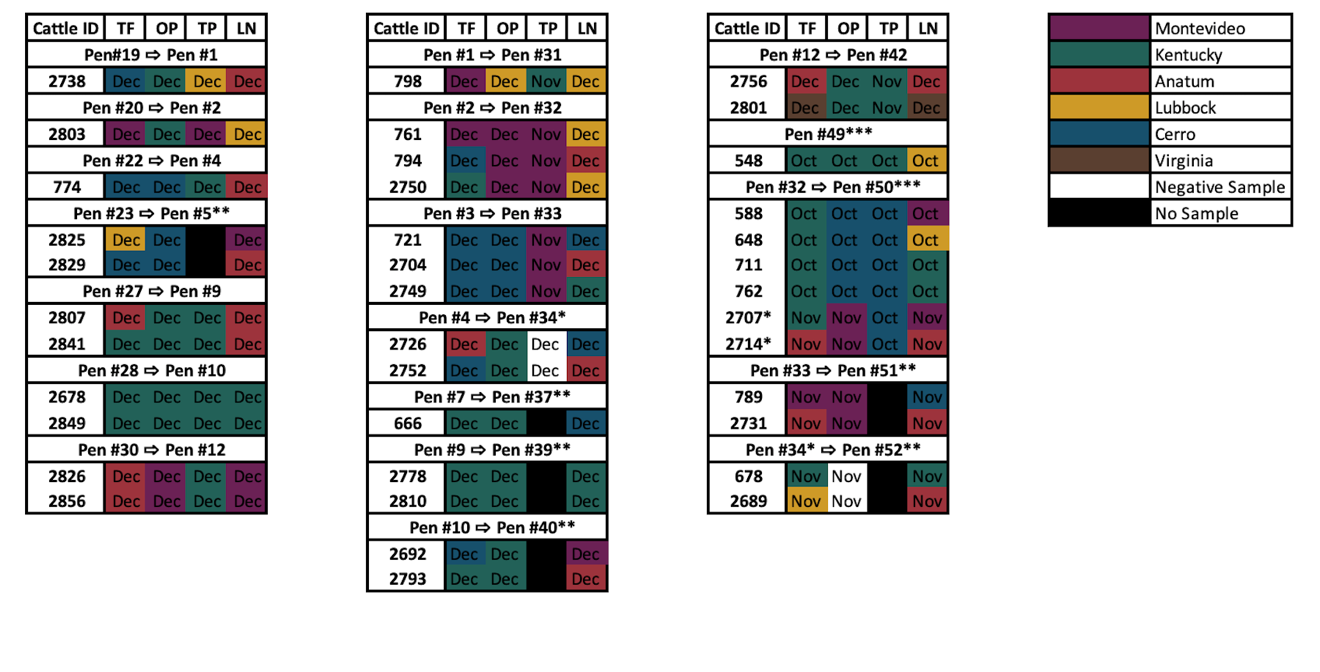


**Figure S1** Serovars of matched samples from individual cattle, identified by cattle ID, subiliac lymph node (LN) isolates, paired with terminal fecal (TF) isolates, terminal pen environment (TP) isolates and original pen (OP) environment isolates. The original pen and terminal pen numbers are indicated at the top of each group of cattle that were contained within those pens. Color coding indicates the Salmonella serovar related to the isolate and within each cell the collection month is included. In some cases, the pen sample was negative as indicated by a white cell; further, there was no sample collected from some pens as indicated by a black cell.


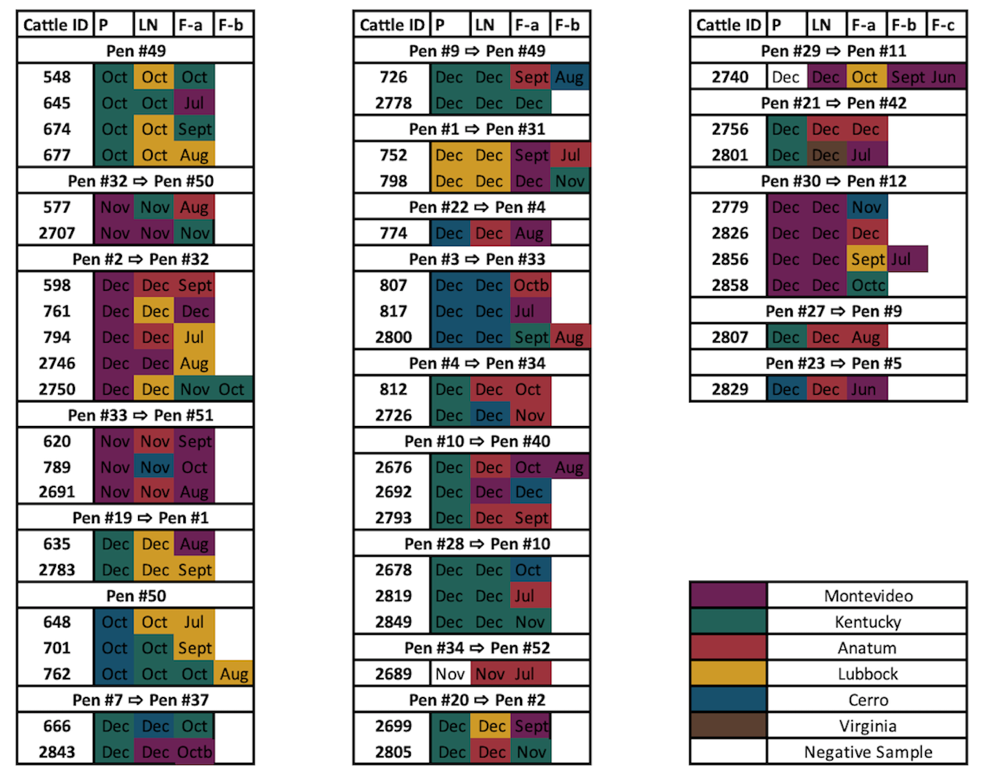


**Figure S2** Non-terminal fecal (F-a, b, c) and pen environmental (P) isolates that paired with lymph node isolates (LN) across the duration of the study. The layout is consistent with **S1**.


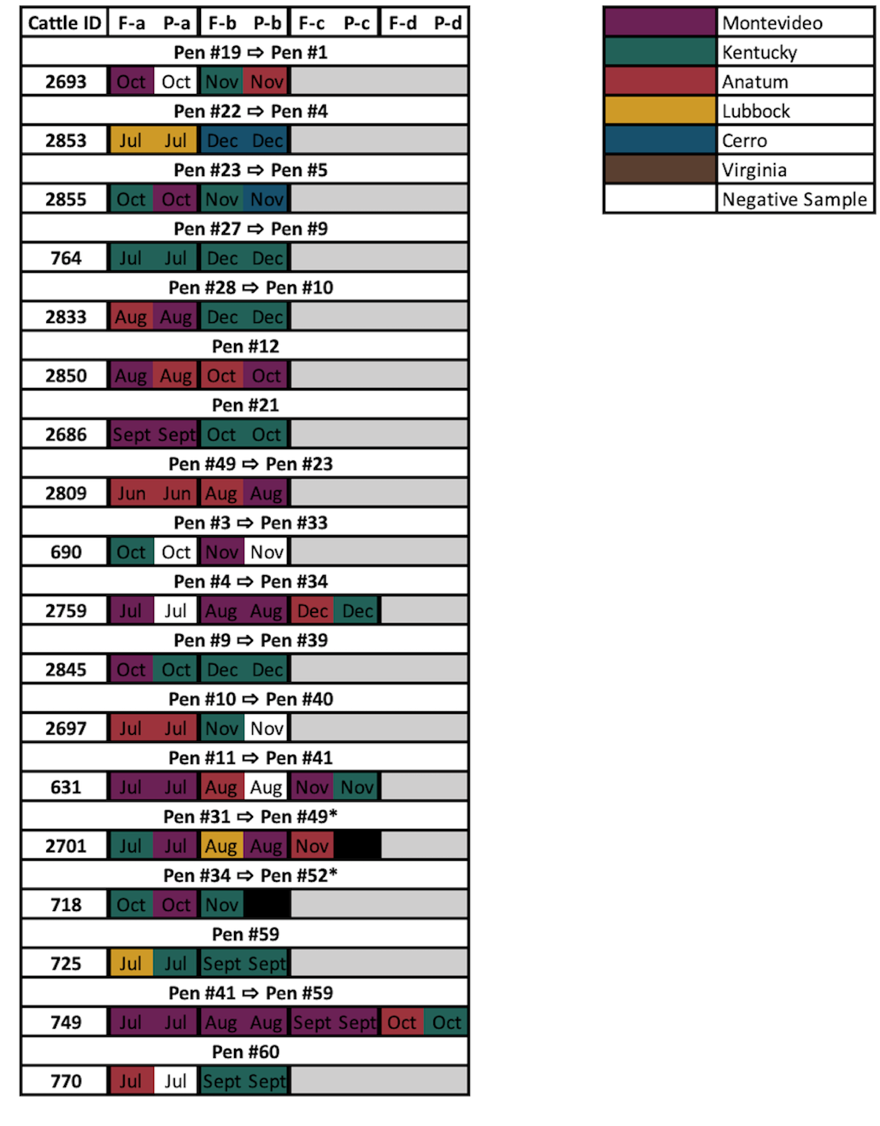


**Figure S3** There were several fecal (F: a, b, c, d) and pen environmental (P-a, b, c, d) isolates related to the same animal, indicated by Cattle ID, during the longitudinal study. *An environmental sample was missing from Pen #49. ** There was a pen (Pen #52) in which the environment was not sampled because it was part of the nutritional study, not the longitudinal study pen. The layout is consistent with **S1** & **S2**.

| ***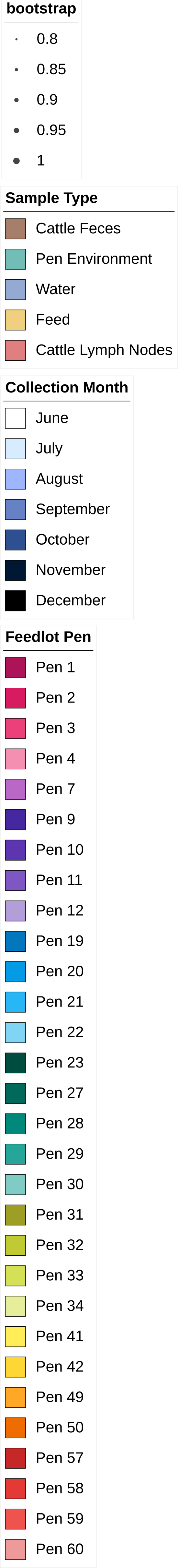*** | ***A***  ***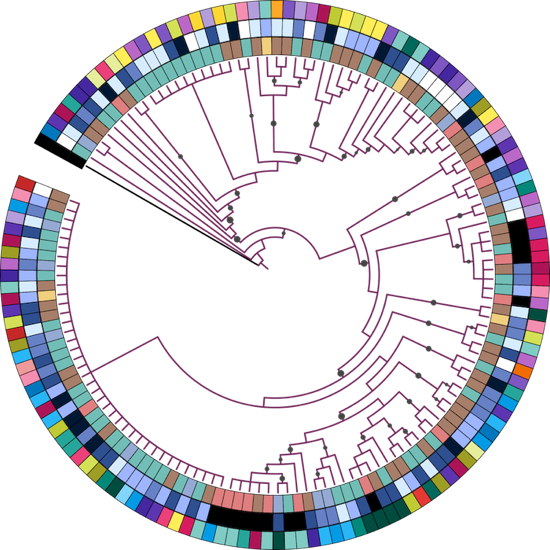*** | ***B***  ***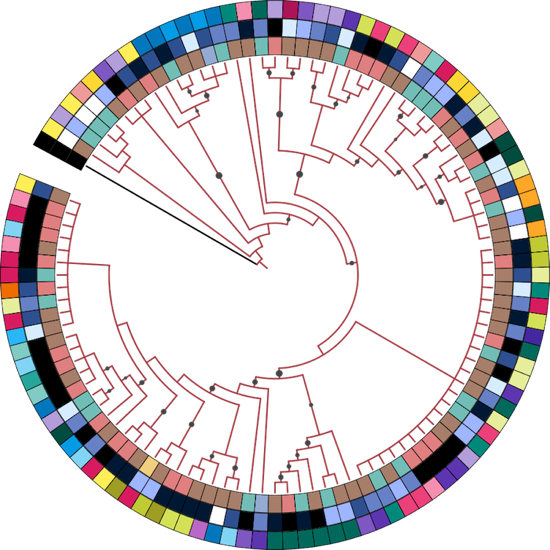*** |
| --- | --- | --- |
|  | ***C***  ***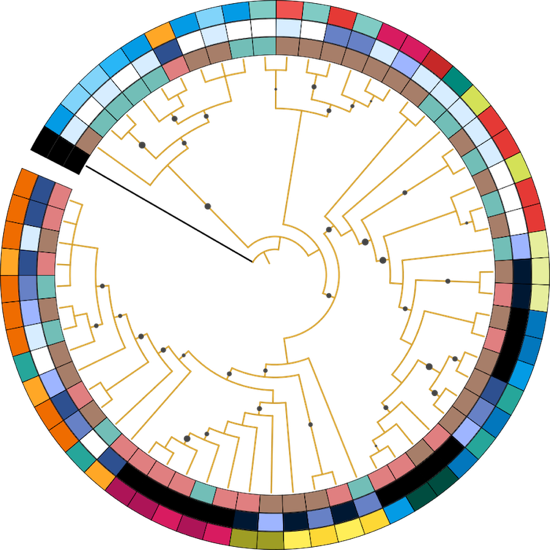*** | ***D***  ***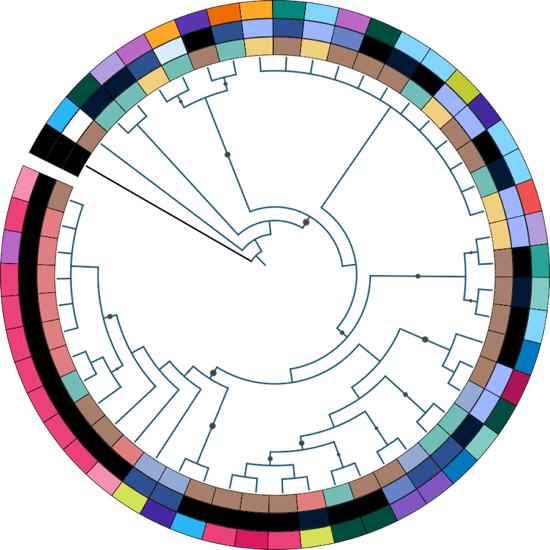*** |
|  | **Figure S4A**) Phylogenetic trees for Salmonella serovars Montevideo isolates, **4B)** serovar Anatum isolates, **4C)** serovar Lubbock isolates, and **4D)** serovar Cerro isolates. All of the phylogenetic trees were set up with metadata from the inner ring out as follows: collection month, sample type, and feedlot pen. Pen is shaded within feedlot blocks. | |
